# Sequential neural dynamics underlie unconscious integration and conscious perception of visual stimuli

**DOI:** 10.1371/journal.pbio.3003894

**Published:** 2026-07-06

**Authors:** Maëlan Q. Menétrey, Michael H. Herzog, David Pascucci

**Affiliations:** 1 Laboratory of Psychophysics, Brain Mind Institute, École Polytechnique Fédérale de Lausanne (EPFL), Lausanne, Switzerland; 2 Psychophysics and Neural Dynamics Lab, Department of Radiology, Lausanne University Hospital (CHUV) and University of Lausanne (UNIL), Lausanne, Switzerland; 3 The Sense Innovation and Research Center, Lausanne, Switzerland; University of Amsterdam: Universiteit van Amsterdam, NETHERLANDS, KINGDOM OF THE

## Abstract

In some forms of postdictive phenomena, later events influence the perception of earlier ones, suggesting that conscious perception may be preceded by extended periods of unconscious processing. An example is the Sequential Metacontrast (SQM) paradigm, in which vernier offsets are unconsciously integrated over several hundred milliseconds before conscious perception. Obviously, the integrated percept can only emerge after each individual element in the stream has been processed. Thus, the SQM provides a unique opportunity to dissociate unconscious from conscious stages of visual processing, as these stages are well separated in time. Using electroencephalography (EEG) recordings in human participants during the SQM, we identified two distinct stages of neural activity: an early occipital EEG activity pattern (~200 ms after the initial vernier) associated with unconscious processing, and a later centro-parietal EEG pattern (~400 to 600 ms after SQM onset) associated with the integrated percept and the behavioral report. We propose that the transition between these neural patterns marks the shift from unconscious encoding of individual visual stimuli to their integrated percept.

## Abstract 2

Understanding the neural correlates of consciousness remains a challenge, as disentangling unconscious from conscious processing is highly complex. Using EEG decoding, this study provides neural evidence supporting a two-stage model of visual processing, with distinct brain topographies marking the shift from the unconscious encoding of individual visual stimuli to their integrated conscious percept.

## Introduction

Perception feels like a continuous stream: we seem to consciously experience the world at each moment of time. However, several visual phenomena challenge this idea. For instance, in a classic apparent motion phenomenon, two dots of different colors are flashed at separate locations with a brief delay [1]. Rather than perceiving two distinct flashes one after the other, we perceive a single moving dot that changes color midway. Logically, the color change can only be perceived after the second dot is presented, implying that later events influence how we perceive earlier ones. These postdictive effects provide a unique opportunity to study the neural correlates of both conscious and unconscious processing, as well as the transitions between the two.

**Fig 1:**
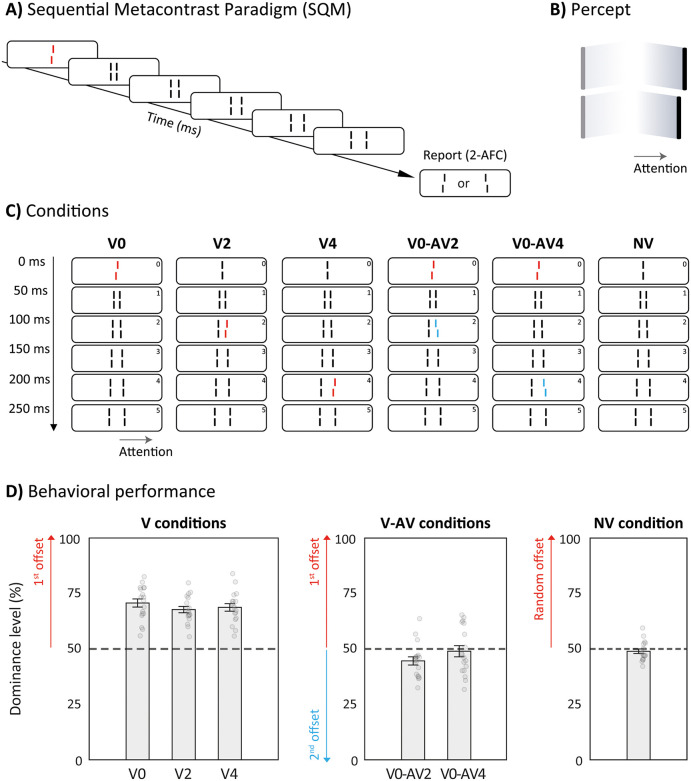
The Sequential Metacontrast (SQM) paradigm. **(A)** A central line, here with a left vernier offset, is followed by pairs of flanking lines eliciting a percept of two diverging streams. Participants attend to one stream (in this study, the rightward stream) and, at the end of each trial, report the perceived offset using a hand-held push-button response (left vs. right, two-alternative forced choice). **(B)** In the attended stream, the offset is typically perceived as propagating along the entire stream. **(C)** In V conditions, only one vernier is presented, either in the central line (V0), in the second (V2), or in the fourth (V4) flanking line. In V-AV conditions, the central line is offset, and one of the flanking lines is offset in the opposite direction (anti-vernier; V0-AV2 or V0-AV4). In a control condition (NV), only straight lines are shown. The red and blue vernier offsets in the Figure are for illustration purposes only; in the experiment, all lines were the same color. **(D)** In V conditions, participants could well report the offset direction. In V-AV conditions, participants could not perceive the individual vernier offsets, and hence performance is at 50%. This indicates that both vernier offsets contribute equally to the integrated percept, or that each offset is reported with equal probability in a given trial. When no vernier was presented or perceived, participants were guessing. In NV condition, performance is determined by comparing the response to a randomly chosen offset. The data underlying Fig 1D can be found in S1 Data.

A prime tool for studying these processes is the Sequential Metacontrast (SQM) paradigm (Fig 1A and 1B). In this paradigm, a sequence of vertical lines is presented, creating the illusion of two diverging motion streams expanding from a central point [2,3]. When one of the lines contains a horizontal vernier offset (i.e., the lower segment of the line is slightly shifted leftward or rightward relative to the upper segment), all the other lines are perceived as having the same offset, even though they are actually straight. When the stream contains two verniers with opposite offsets, the offsets integrate: participants are unable to report the individual offsets separately and cannot locate which lines carry offsets [4]. Thus, the multiple offsets are integrated unconsciously and the result of the integration is the final conscious percept.

In the SQM, the integration period of the vernier offsets is approximately 290–450 ms, depending on the participant and the condition [5,6]. When two offsets are separated by more than ~450 ms, participants can report the offsets individually [4,5]. These findings suggest that the brain integrates features within a relatively long-lasting window of unconscious processing, followed by an integrated conscious percept [7,8]. The existence of such long-lasting integration windows has been demonstrated not only in visual perception [9–13], but also in auditory perception [14] and across different sensory modalities [15–17].

In this study, we used electroencephalography (EEG) decoding to investigate the neural mechanisms involved in both the unconscious integration of vernier offsets and the subsequent emergence of a conscious percept. Our approach was guided by the following rationale: in the SQM, regardless of the number and spatiotemporal locations of the verniers, all offsets are *integrated unconsciously*, and only the outcome is *consciously perceived* [8]. Neural activity encoding information about the number or location of individual offsets should therefore reflect unconscious processing, whereas neural activity encoding the integrated outcome should reflect processes linked to the content of the conscious percept.

## Results

### Unconscious feature integration within the SQM stream

In the conditions with only a single vernier in the stream (V conditions; Fig 1A and 1C), offset discrimination was above 50% (Fig 1D, left panel). This effect was consistent regardless of where the offset appeared in the stream: whether the offset occurred in the central line or in the second or fourth flanking lines, mean accuracy remained high (70% ± 7%). Paired t-tests comparing each of these V conditions with a no vernier (NV) control condition resulted all in significant differences (all *p* < .001, Cohen’s *d′* > 3.3; Fig 1D, left panel; S1 Data).

In contrast, when two opposite vernier offsets (a vernier and an anti-vernier) were included in the stream (V-AV condition, Fig 1C), mean accuracy was 47% ± 9%, comparable to the NV condition (paired t-tests between the V-AV and NV conditions: all *p* > .05; Fig 1D, right panel; S1 Data). Thus, conscious perception does not depend on the spatiotemporal location and number of offsets in the stream, as there is no conscious percept of the individual elements.

### Neural representations preserve the chronology of events in the SQM stream

We first aimed to decode neural activity related to the processing of individual vernier offsets. To this end, we applied Linear discriminant analysis (LDA) to EEG activity patterns to decode the presence and spatiotemporal location of vernier offsets across the stream.

**Fig 2:**
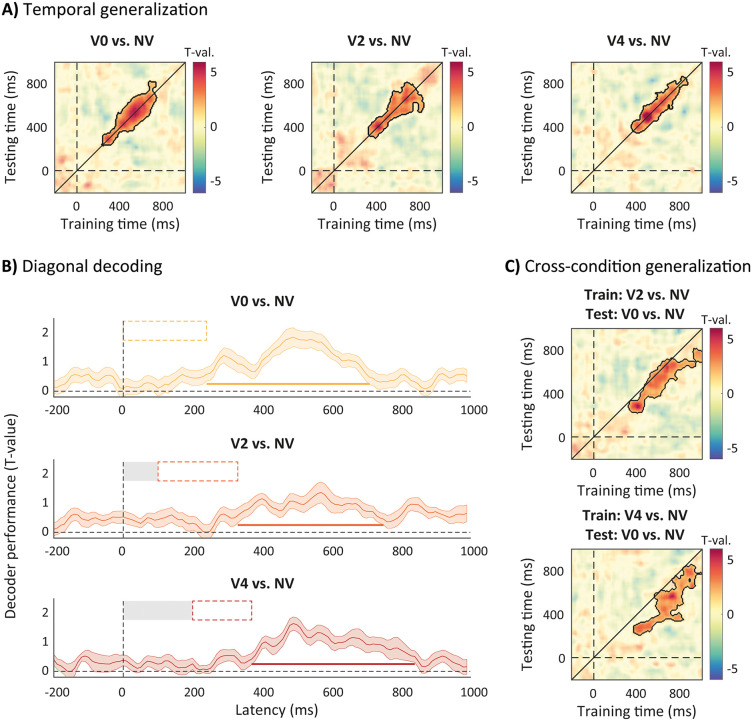
Linear discriminant analysis (LDA) with temporal and cross-condition generalization to decode conditions with one vernier and no vernier. **(A)** Temporal generalization matrices, obtained by training a classifier on data at every time point and testing it at all other time points. The classifiers can successfully discriminate between the conditions (V0, V2, and V4 vs. NV). Significant clusters are highlighted (AUC > 0.5, one-tailed cluster-based permutation test, *p* < .05). **(B)** Diagonal elements of the temporal generalization matrices, representing decoding results when training and testing at the same time point (group average AUC and SEM). Significant time windows are highlighted by the horizontal lines at the bottom. Temporal delays in the decoding results, relative to the actual onset of the vernier in the stream, are indicated by the dashed rectangles. **(C)** Cross-condition generalization matrices showing that classifiers trained with V2 or V4 vs. NV successfully discriminate V0 vs. NV but with earlier onsets corresponding to the effective stimulus delay between V0 and V2 (i.e., 100 ms later) and V4 (i.e., 200 ms later). Significant clusters are highlighted (AUC > 0.5, one-tailed cluster-based permutation test, *p* < .05). The data underlying this Figure can be found in https://doi.org/10.5281/zenodo.20729504.

We ran separate LDA classifiers to discriminate between trials with no offset (NV condition) and trials with a single vernier offset presented at different spatiotemporal locations within the stream (V0: 0 ms, V2: 100 ms, V4: 200 ms, Fig 1C). Using a temporal generalization approach, the classifiers were trained on data from each time point and tested across all other time points (see Materials and methods). The classifiers successfully discriminated the presence of the vernier offsets in all V conditions (area under the curve [AUC] > 0.5, one-tailed cluster-based permutation test, *p* < .05; Fig 2A and 2B). The resulting patterns were similar across conditions with decoders generalizing only over short time windows, as indicated by higher decoder performance when both training and testing occurred at the same time points (i.e., the diagonal elements of the temporal generalization matrices).

Crucially, the decoding latencies revealed systematic delays that corresponded to the actual spatiotemporal location of the vernier offset in the stream—decoding was successful at 240 ms for V0 versus NV, at 330 ms for V2 versus NV, and at 370 ms for V4 versus NV (Fig 2B). Hence, although participants perceived the offset in the entire stream, the offset was decoded only around 200 ms (170–240 ms) after its physical onset, with slightly decreasing delays when the vernier was presented later in the stream.

This result was further supported by cross-condition decoding, where we trained a classifier on discriminating V2 versus NV or V4 versus NV and tested it on V0 versus NV. This revealed shared neural representations of the single vernier across conditions but shifted along the diagonal in the cross-condition temporal generalization matrix (Fig 2C). That is, a decoder trained to detect the vernier in the V2 or V4 condition successfully decoded it in V0 (AUC > 0.5, one-tailed cluster-based permutation test, *p* < .05), but with earlier onsets of approximately 100 and 200 ms, respectively, corresponding to the actual latencies between conditions.

**Fig 3:**
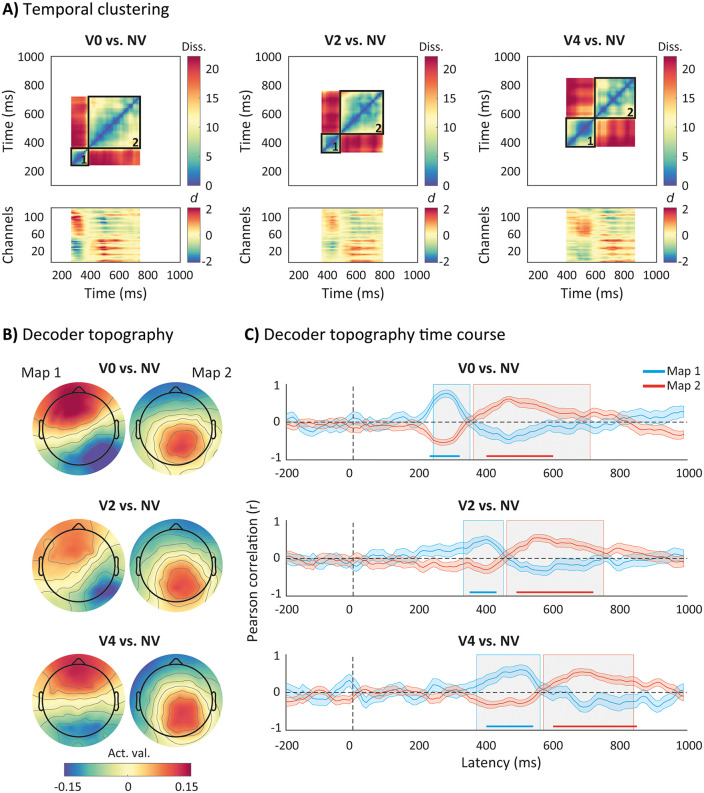
EEG activation patterns contributing to decoding conditions with one vernier and no vernier. **(A)** Analysis of dissimilarity matrices between the EEG activation patterns extracted from significant time points (diagonal elements of the temporal generalization matrices for V0, V2, or V4 vs. NV; see Fig 2B) reveals a clear transition between two main activation patterns (outlined by black squares) for each decoding analysis (upper row). These activations patterns were consistently expressed across participants, with many channels and time points showing large effect sizes (difference between each activation pattern and zero, calculated for each electrode and time point |Cohen’s d| > 1; bottom row). **(B)** Decoder topographies, averaged across participants, are derived from the two main activations patterns identified with the analysis of dissimilarity matrices. Each decoding analysis shows similar topographical maps but with variations in their transition dynamics. **(C)** Temporal correlations between the two identified topographies and the back-projected time course of EEG activation patterns for each decoding analysis. Gray areas highlight the significant decoding windows found in Figs 2B and 3A. Blue and red lines represent the occipital and parietal topographies, respectively, and the shaded areas indicate SEM. Significant positive correlations are also highlighted in blue for the occipital topography or in red for the parietal topography (Pearson’s *r* > 0, one-tailed cluster-based permutation test, *p* < .05). The data underlying this Figure can be found in https://doi.org/10.5281/zenodo.20729504.

Decoding the conditions with a single vernier against the condition with no vernier was significant in windows ranging from 240 to 710 ms in V0 versus NV, 330–750 ms in V2 versus NV, and 370–840 ms in V4 versus NV). In these time windows, we found two distinct EEG activation patterns, i.e., topographies of EEG activity associated with the decoder results (see Materials and methods and Fig 3A). These scalp patterns and their transitions were consistent across participants (Fig 3A, bottom row, Cohen’s *d* of the scalp activation patterns at the group level).

The first topography displayed a stronger contribution of occipital electrodes (hereafter referred to as the “occipital topography”), with a duration that increased as the vernier offset occurred later in the stream (Fig 3B). In contrast, the second topography showed a stronger contribution of parietal electrodes (hereafter referred to as the “parietal topography”), with a duration that decreased as the vernier offset occurred later (Fig 3B). The time course of these topographies, estimated by back-projecting activation patterns onto the original EEG time series (see Materials and methods), revealed a clear transition between the two patterns, with evident temporal shifts across conditions (i.e., the transition occurred around 360 ms in V0 versus NV, 460 ms in V2 versus NV, and 570 ms in V4 versus NV; Fig 3C). We further confirmed the robustness of the effect across participants by estimating the proportion of individual activation patterns showing a positive correlation with the occipital topography within the time window corresponding to the first cluster (17/18 participants in V0 versus NV, 15/18 in V2 versus NV, and 15/18 in V4 versus NV), as well as those showing a positive correlation with the parietal topography within the time window corresponding to the second cluster (17/18 participants in V0 versus NV, 15/18 in V2 versus NV, and 17/18 in V4 versus NV).

### Neural correlates of unconscious processing

Up to this point, we have focused on conditions featuring a single vernier, whose offset is consciously perceived throughout the entire stream. Even though the spatiotemporal location of the vernier was not consciously perceived, it could still be decoded via two distinct EEG topographies. Next, we analyzed conditions involving two opposite vernier offsets, separated by varying intervals (V0-AV2: 50 ms, V0-AV4: 150 ms; Fig 1C), using LDA to distinguish these conditions from a condition featuring only straight lines (NV).

In the V-AV conditions, behavioral performance was around 50% (Fig 1D). As previously suggested, this may reflect either that both offsets are fully integrated, resulting in the perception of a straight line as in the NV condition [4], or that there is an equal probability that one of the two vernier offsets ”wins” the integration process in a given trial [18].

**Fig 4:**
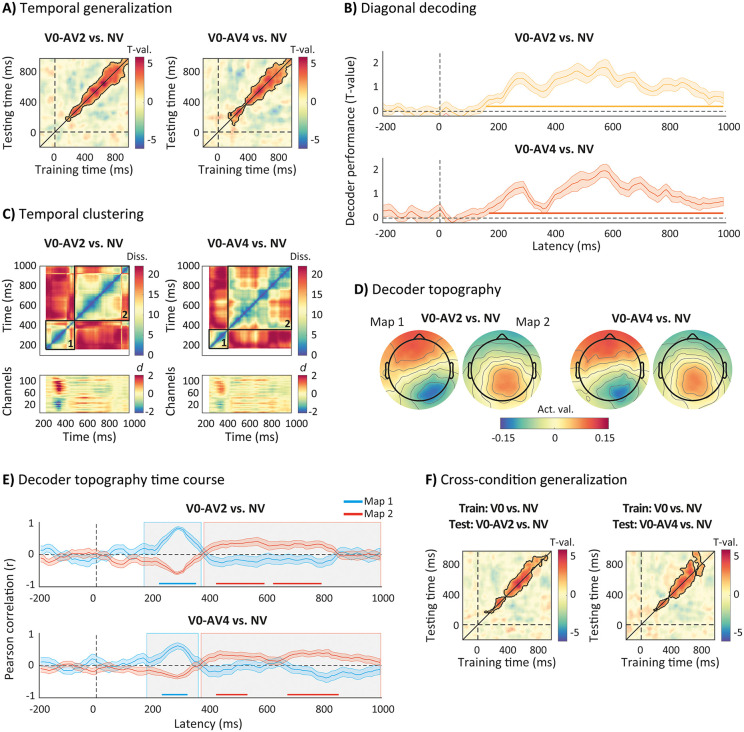
LDA with temporal and cross-condition generalization to decode conditions with two opposite verniers and no vernier. **(A)** Temporal generalization matrices showing that classifiers successfully discriminate the conditions (V0-AV2 or V0-AV4 vs. NV). Significant clusters are highlighted (AUC > 0.5, one-tailed cluster-based permutation test, *p* < .05). **(B)** Diagonal elements of the temporal generalization matrices for V0-AV2 or V0-AV4 vs. NV, representing decoding results when training and testing at the same time point (group average AUC and SEM). Significant time windows are highlighted by the horizontal lines at the bottom. **(C)** Analysis of dissimilarity matrices between the EEG activation patterns extracted from significant time points reveals a clear transition between two main activation patterns (outlined by black squares) for each decoding analysis (upper row). These activations patterns were consistently expressed across participants, with many channels and time points showing large effect sizes (difference between each activation pattern and zero, calculated for each electrode and time point |Cohen’s d| > 1; bottom row). **(D)** Decoder topographies, averaged across participants, are derived from the two main activation patterns identified with the analysis of dissimilarity matrices. Each decoding analysis shows similar topographical maps and transition dynamics. **(E)** Temporal correlation between the two identified topographies and the back-projected time course of EEG activation patterns for each decoding analysis. Gray areas highlight the significant decoding windows found in Fig 4B and 4C. Blue and red lines represent the occipital and parietal topographies, respectively, and the shaded areas indicate SEM. Significant positive correlations are also highlighted in blue for the occipital map or in red for the parietal (Pearson’s *r* > 0, one-tailed cluster-based permutation test, *p* < .05). **(F)** Cross-condition generalization matrices showing that classifiers trained with V0 vs. NV successfully discriminate V0-AV2 or V0-AV4 vs. NV with similar onset. Significant clusters are highlighted (AUC above 0.5, one-tailed cluster-based permutation test, *p* < .05). The data underlying this Figure can be found in https://doi.org/10.5281/zenodo.20729504.

The decoder successfully discriminated V0-AV2 or V0-AV4 trials from NV trials (AUC > 0.5, one-tailed cluster-based permutation test, *p* < .05; Fig 4A). In both analyses, the significant decoding windows (170–1,000 ms; Fig 4B) exhibited a similar sequence of EEG activation patterns (Fig 4C), with topographies (Fig 4D) closely resembling those observed in the V0 versus NV decoding analysis (Fig 3B). Similarly, the significant decoding onsets and the transition dynamics around 370 ms (Fig 4B and 4E) closely mirrored those found in V0 versus NV conditions (Figs 2B and 3C for comparison). The effect was also robust across participants (positive correlation with the occipital topography within the time window associated with the first cluster: 18/18 participants in V0-AV2 versus NV and 14/18 in V0-AV4 versus NV; positive correlation with the parietal topography within the time window associated with the second cluster: 16/18 participants in V0-AV2 versus NV and 18/18 in V0-AV4 versus NV). Thus, EEG activation patterns distinguished the presence of two offsets from no offsets, even though the individual offsets were integrated and not perceived separately.

Furthermore, cross-condition decoding revealed that a decoder trained to detect the vernier in the V0 condition (versus NV) still successfully decoded, with similar onset and temporal dynamics, V0-AV2 or V0-AV4 conditions from NV condition (AUC > 0.5, one-tailed cluster-based permutation test, *p* < .05, Fig 4F), indicating shared neural representations between conditions with a single vernier and two opposite vernier offsets.

**Fig 5:**
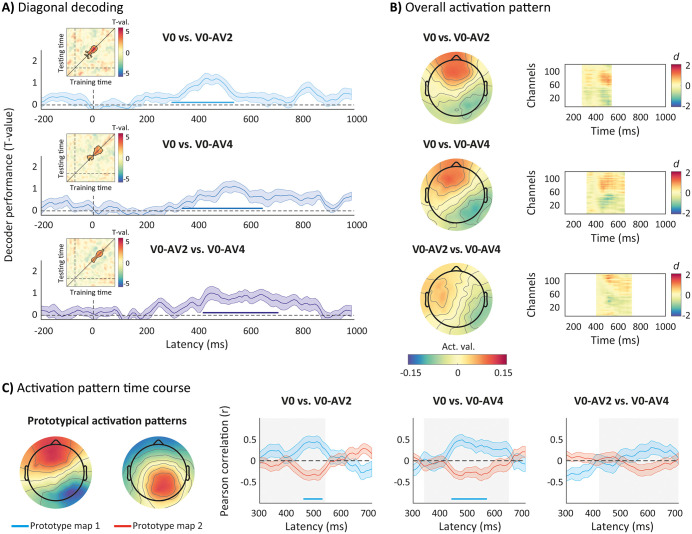
LDA between conditions with one and two verniers and between conditions with two verniers. **(A)** Diagonal elements of the temporal generalization matrices (shown in the insets), representing decoding results when training and testing at the same time point (group average AUC and SEM). Significant time windows are highlighted by the horizontal lines at the bottom, showing that classifiers successfully discriminate the conditions (V0 vs. V0-AV2 or V0-AV4, and V0-AV2 vs. V0-AV4; AUC > 0.5, one-tailed cluster-based permutation test, *p* < .05). **(B)** The decoder topographies, averaged across participants, are derived from the averaged activation pattern over the entire significant window of each decoding analysis. These activations patterns were consistently expressed across participants, with many channels and time points showing large effect sizes (difference between each activation pattern and zero, calculated for each electrode and time point |Cohen’s *d*| > 1; bottom row). **(C)** Prototypical activation patterns (left side) reflecting the occipital (map 1) and parietal topographies (map 2), corresponding to the average of the two distinct activation patterns identified via dissimilarity matrix analysis for all V or V-AV vs. NV contrasts (see Figs 3B and 4D), were temporally correlated with the activation patterns found in each decoding window (right side). Gray areas highlight the significant decoding window found in Fig 5A. Blue and red lines represent the occipital and parietal topographies, respectively, and the shaded areas indicate SEM. Significant positive correlations are also highlighted in blue for the occipital topography or in red for the parietal topography (Pearson’s *r* > 0, one-tailed cluster-based permutation test, *p* < .05). The data underlying this Figure can be found in https://doi.org/10.5281/zenodo.20729504.

Importantly, we also found that the two V-AV conditions, where the second vernier occurred at different times (V0-AV2 versus V0-AV4), could be decoded from V0 trials, where only a single vernier was shown and consciously perceived (AUC > 0.5, one-tailed cluster-based permutation test, *p* < .05; Fig 5A). The corresponding decoding windows (from 300 ms for V0-AV2 versus V0, and from 340 ms for V0-AV4 versus V0) were compatible with the latencies observed when decoding conditions in which a single vernier was physically presented later in the stream (V2 or V4 versus NV, Fig 2B). Similarly, the two V-AV conditions were also reliably discriminated from each other (AUC > 0.5, one-tailed cluster-based permutation test, *p* < .05; Fig 5A). That is, EEG activation patterns contained information about the distinct spatiotemporal location of the second vernier, which was decodable starting from 420 ms onward. These results suggest that neural representations of the second vernier persisted despite the second offset not being typically consciously perceived as a distinct event at its specific spatiotemporal location [4,19].

The average activation pattern contributing to these decoding results (Fig 5B) resembled the occipital topography identified when contrasting conditions with one or two offsets from the condition without any offset (Figs 3B and 4D). This topography dominated and remained stable throughout the significant decoding windows and across the three analyses (V0 versus V-AV and V0-AV2 versus V0-AV4; see Fig 5B, effect size plots). To further validate this finding, we correlated the activation patterns obtained here with the two prototypical topographies derived from averaging across all V or V-AV versus NV decoding analyses (Figs 3B and 4D; see Materials and methods). The results confirmed the dominant contribution of the occipital prototypical topography (Fig 5C), temporally aligned with the onset of the second vernier. We further corroborated the dominant occipital contribution by decoding between V conditions involving physically different sequences of events that nevertheless led to the common conscious perception of a single integrated offset (V0 versus V2 or V4, and V2 versus V4). These results further suggest that this topography reflects the processing of the spatiotemporal location of a vernier offset prior to its integration within the SQM stream (see S1 Text and S1 Fig).

Based on these findings, we interpret the occipital topography as the neural correlate of unconscious processing of the vernier stimuli, occurring prior to the formation of the integrated percept. Conversely, the presence of the parietal topography when discriminating V-AV from NV trials (Fig 4D) might reflect the content of the final integrated percept. Indeed, the latencies and sequence of topographies in these decoding analyses are similar to those observed in the condition with a single vernier presented in the central line (V0; Fig 3B), consistent with the view that, in V-AV conditions, participants may still perceive a residual offset in the stream [18]. Such a percept could arise from a non-uniform integration of the first and second vernier offsets, resulting in one offset contributing more strongly than the other (see S1 Text and S2 Fig for additional cross-condition generalization evidence between V and V-AV conditions, as a function of whether the first or second vernier dominated the reported percept).

### Neural correlates of the integrated percept

**Fig 6:**
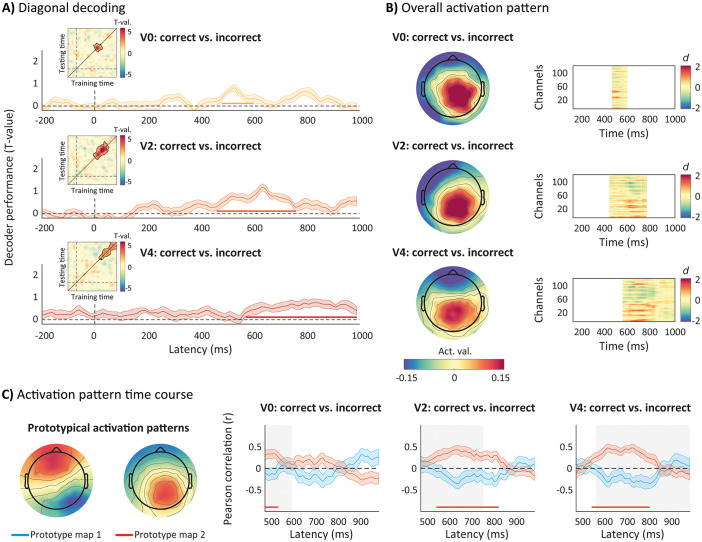
LDA between correct and incorrect reports in conditions with one vernier. **(A)** Diagonal elements of the temporal generalization matrices (shown in the insets), representing decoding results when training and testing at the same time point (group average AUC and SEM). Significant time windows are highlighted by the horizontal lines at the bottom, showing that classifiers successfully discriminate correct from incorrect offset reports in all V conditions (V0, V2 or V4; AUC > 0.5, one-tailed cluster-based permutation test, *p* < .05). **(B)** The decoder topographies, averaged across participants, are derived from the averaged activation pattern over the entire significant window of each decoding analysis. These activations patterns were consistently expressed across participants, with many channels and time points showing large effect sizes (difference between each activation pattern and zero, calculated for each electrode and time point |Cohen’s d| > 1; bottom row). **(C)** Prototypical activation patterns (left side) reflecting the occipital (map 1) and parietal topographies (map 2), corresponding to the average of the two distinct activation patterns identified via dissimilarity matrix analysis for all V or V-AV vs. NV contrasts (see Figs 3B and 4D), were temporally correlated with the activation patterns found in each decoding window (right side). Gray areas highlight the significant decoding window found in Fig 6A. Blue and red lines represent the occipital and parietal topographies, respectively, and the shaded areas indicate SEM. Significant positive correlations are also highlighted in blue for the occipital topography or in red for the parietal one (Pearson’s *r* > 0, one-tailed cluster-based permutation test, *p* < .05). The data underlying this Figure can be found in https://doi.org/10.5281/zenodo.20729504.

To directly assess the involvement of the parietal topography in conscious processing, we applied LDA to decode correct versus incorrect reports of the offset direction in the conditions featuring only a single vernier (V conditions). The classifiers successfully decoded the performance in all single-vernier conditions (Fig 6A; AUC > 0.5, one-tailed cluster-based permutation test, *p* < .05). However, significant decoding emerged at later latencies, 480 ms for V0, 460 ms for V2, and 570 ms for V4, compared to the earlier latencies observed when decoding V or V-AV conditions against the NV condition.

In addition, the topographies obtained by averaging the main activation patterns across the entire significant decoding window (Fig 6B) were clearly distinct from those identified in the previous decoding analyses (V0 versus V-AV and V0-AV2 versus V0-AV4; Fig 5B). Correlations with prototypical activation patterns confirmed that decoding of the correctly reported direction was driven by a single EEG activation pattern (Fig 6C), which in this case aligned with the parietal topography identified when decoding conditions with one or two offsets from trials without any offset (Figs 3B and 4D). This topography exhibited latency shifts as a function of the vernier’s temporal position within the stream and appeared to persist longer when the vernier occurred later in time (see S1 Text and S2 Fig for analogous effects in V–AV conditions, depending on whether the first or second vernier dominated the integrated percept).

Thus, the later decoding of correct versus incorrect responses, compared to the earlier decoding reflecting differences in the spatiotemporal location or number of verniers in the stream, indicates that this centro-parietal topography reflects a neural correlate of a later stage, involving processes associated with the consciously perceived vernier offset, rather than the initial stage of unconscious integration.

## Discussion

Previous studies have proposed that in the SQM, and in related postdictive phenomena, conscious perception is preceded by a period of unconscious processing [7,8,13,20–23]. In the SQM, a sequence of lines and vernier offsets is unconsciously integrated for up to 450 ms before a conscious percept emerges [4,5]. Here, we used time-resolved decoding analysis to identify EEG markers of this unconscious integration stage and to track the transition toward the final integrated percept.

We found that each vernier offset in the stream could be decoded from EEG activity time-locked to the moment it was shown, with decoding emerging around 200 ms later (Fig 2). This was the case even though the consciously perceived vernier offset does not depend on the number or specific spatiotemporal location of the verniers within the SQM stream. Importantly, individual verniers remained decodable even when two opposite verniers were presented and their offsets were integrated (Fig 4). These results indicate that, even when individual vernier offsets are not consciously perceived, their presence and temporal position can still be inferred from neural activity.

The decoding of individual vernier offsets was driven predominantly by occipital activity (see Figs 3 and 4), with a topography resembling the N170/VAN ERP component (see also S1 Text and S3 Fig for complementary ERP analyses). Although this component has often been proposed as an early correlate of conscious perception [24–27], several studies have related it to response to unseen stimuli [28–30]. Our results suggest that the N170/VAN may reflect an unconscious rather than a conscious processing stage, consistent with studies linking this component to the unconscious accumulation and integration of visual information [31,32].

The N170/VAN-like component was also observed when decoding between conditions involving one or two verniers (Fig 5), even though both conditions produced the same integrated percept, namely, a single vernier offset perceived throughout the stream. Because these conditions differed in the location or number of physical offsets, this finding suggests that the topography reflects differences in the sequence of unconsciously processed events, supporting its role as a neural correlate of unconscious integration. It is also worth noting that this topography was right-lateralized (ipsilateral to the stimulus). The origin of this lateralization remains unclear, and future studies should assess the effect of attending to the left versus the right stream, as participants in the present study attended only to the right stream. One possibility is that the right hemisphere dominates processing of motion-related signals from both visual hemifields [33,34], or, more generally, that it reflects a right-hemisphere specialization for visuo-spatial processing [35].

Following this occipital topography, we identified a distinct parietal topography (see Figs 3 and 4). This parietal topography contributed to decoding conditions with one or more verniers against the condition with no vernier, but it did not emerge when decoding between conditions containing one versus two verniers. This suggests that it carries information about the integrated percept, regardless of whether it arises from a single vernier or from the integration of two. Consistent with this interpretation, a similar centro-parietal topography was observed when decoding correct versus incorrect behavioral reports of the offset in conditions with a single vernier (see Fig 6). This topography emerged after approximately 450 ms and persisted for several hundred milliseconds, resembling the P300 ERP component (see also S1 Text and S3 Fig for complementary ERP analyses).

The P300 is often described as a neural correlate of conscious access [32,36–40] and has been linked to late-access models of consciousness, where conscious perception arises from higher-order, distributed or recurrent neural processes [41–46]. However, recent studies suggest that, rather than reflecting the neural correlates of conscious perception, the P300-like component may index post-perceptual processing stages, such as decision-making and response-related activity, because it is often absent in no-report paradigms or for task-irrelevant but consciously perceived stimuli [47–50]. In our task, a direct motor contribution can be ruled out, as responses occurred much later, around 900 ms. Nevertheless, a post-perceptual interpretation remains possible, and future studies incorporating no-report paradigms will be important to further clarify the role of this component.

Here, we suggest that the critical shift from unconscious processing to the unified integrated percept is not reflected in the P300-like component itself, but rather in the transition from the occipital to the parietal topography. This interpretation is further supported by the close temporal alignment between this transition (Figs 3 and 4) and the window of unconscious integration previously estimated from behavioral measures (~290 to 450 ms; [4,5].

Thus, our results support the existence of long-lasting unconscious processing windows, which have been reported in other postdictive paradigms beyond the SQM [11,51–54], and even with briefly presented stimuli. For example, when a 30 ms vernier is followed by a 30 ms anti-vernier, participants perceive a single integrated vernier without any percept of apparent motion [9]. Yet, transcranial magnetic stimulation (TMS) can alter this integrated percept up to 360 ms after stimulus onset, shifting dominance toward one of the two initial verniers depending on the timing of the TMS pulse [9]. These findings collectively support the idea that unconscious integration can persist well beyond the physical presence of the stimulus (see also Pilz and colleagues [55] for similar results with other visual features than vernier offsets).

Although the unconscious integration window appears relatively long in the SQM, consistent with the fact that individual vernier offsets are not perceived as separate events, its duration may also reflect the demands of motion processing, whereas in other tasks shorter integration windows may allow conscious perception to emerge earlier. Indeed, our results show that the temporal dynamics of unconscious and conscious processing stages are not fixed and may vary even within the same paradigm. For example, when decoding conditions with a single vernier against conditions without any vernier (Figs 2 and 3), the latency between the vernier presentation and the onset of significant decoding decreased when the vernier appeared later in the stream (from 240 ms in V0 versus NV to 170 ms in V4 versus NV; Fig 2B). In these conditions, the occipital topography persisted for a longer duration, whereas the parietal one was shortened (Fig 3A). Moreover, the onset and duration of the second topography appeared to depend on the spatiotemporal location of the vernier dominating the unconscious integration process (Fig 6A; see also S2 Fig). One possibility is that the occurrence of a vernier offset initiates an unconscious processing window, while subsequent offsets or the termination of the SQM stream may accelerate the collapsing of the sequence into a unified conscious percept [56]. Although the exact nature of these non-linearities remains unclear, our results indicate that the temporal dynamics of unconscious and conscious processing stages are flexible and may be influenced by subtle details of the stimulus sequence, in contrast to standard ERP analyses, which often treat processing latencies as fixed.

In general, the fact that neural activity is time-locked to a stimulus (see Fig 2) does not imply that conscious perception occurs at that moment (the ‘vehicle problem’, [8,57–59]), just as a neuron that encodes “red” is not itself red. This contrasts with theories assuming a close mapping between stimulus timing and the moment of perception (e.g., the ‘brain-time’ view, [60]) and implies that neither the transition between the two topographies nor the duration of the parietal topography necessarily reflects neural events involved in conscious perception itself. Instead, these neural signatures may act as precursors to consciousness or, as mentioned, reflect post-perceptual processes operating on the content of the percept. Nevertheless, this transition marks the shift from the unconscious encoding of the physical, retinotopic properties of the vernier offset, which are not perceived consciously, to the integrated representation of the stream, which forms the basis of conscious perception in the SQM. Thus, while the exact timing of conscious perception cannot be inferred directly from these topographies, they reveal the sequential neural processes that enable the emergence of a coherent percept.

In line with this, throughout the manuscript, we use the terms conscious processing and conscious perception to refer to the stage in which an integrated percept of the entire SQM stream has emerged. These terms are used in contrast to the unconscious processing of individual offsets in the SQM, which participants cannot perceive individually. We must clarify, however, that by these terms we do not intend to imply or distinguish between phenomenal consciousness (i.e., the felt quality of a conscious mental state) and access consciousness (i.e., the functions enabled by conscious processing, such as behavioral reports), as our paradigm is not designed to address this distinction [61,62]. As mentioned, we use the terms broadly to include also post-perceptual, decision-making processes associated with the emergence of an integrated percept.

The presence of two distinct topographies, and their temporal dynamics, is compatible with a recently proposed two-stage model of perception in which a prolonged period of unconscious processing precedes the emergence of a unified conscious percept [7,8]. In this framework, the first stage involves the precise encoding of each vernier offset and its temporal position, with this information maintained in a high-capacity buffer where integration operates unconsciously. This stage aligns with the independent neural encoding of individual verniers observed in our decoding results and reflected in the occipital topography (Figs 3–5). In the second stage, the unconscious processing window enables a form of sense-making: the brain resolves the buffered information into the most coherent percept over the preceding hundreds of milliseconds. At the end of this stage, the buffered information collapses into a conscious percept that reflects the integrated structure of the sequence. This transition, and the emergence of a unitary percept, align with the decoding results associated with the parietal topography (Figs 3, 4, and 6). Overall, these sequential stages are consistent with discrete retentional models in which perception is momentary but temporally rich, delayed relative to stimulus onset, yet encoding quantitative details such as duration, speed, and direction [7]. Such dynamics do not contradict the existence of rapid visual detection [63] or the effects of masking [64]. Detection, object recognition, and even action can unfold during unconscious processing without immediately giving rise to conscious awareness ([65–67]; but see also [68,69]).

While many previous findings support the long-lasting unconscious integration of visual features in the SQM [3–6,19,70], the typical use of a forced-choice task based on the offset direction of the first vernier, as employed here, does not allow one to directly infer the exact percept experienced by the participants in the V–AV condition. That is, one cannot infer whether participants perceive a perfectly straight line or a weaker residual offset resulting from non-uniform integration. A recent study showed that the reported offset direction in V–AV conditions, whether consistent with the first or the second vernier, can be predicted by pre-stimulus EEG fluctuations in the alpha band [18]. This finding suggests that integration may indeed vary across trials, with fluctuations in the relative dominance of the first or second vernier. Importantly, regardless of whether integration is uniform or non-uniform, our results show that the spatiotemporal location of each individual vernier, although not consciously accessible as a separate event because all elements are integrated within the SQM stream, can nevertheless be decoded from a specific occipital topography. Considering the role of pre-stimulus oscillatory activity, future studies could further investigate the contribution of specific frequency bands to the unconscious and conscious stages involved in SQM processing, as the present analyses focused exclusively on ERP-based decoding.

In sum, our findings support the presence of sequential processing stages in the SQM and related phenomena, with an extended period of unconscious visual processing followed by a transition to a unified, conscious percept. This has direct relevance for efforts to identify the neural correlates of conscious perception, as most theoretical frameworks, except for the global neuronal workspace theory [42,44], do not explicitly separate unconscious from conscious processing stages (for reviews, see [71,72]). Acknowledging this two-stage structure may therefore provide a useful foundation for future research on the temporal organization of conscious and unconscious visual processing.

## Materials and Methods

### Ethics statement

The experiment was conducted with the approval of the local ethics commission (Commission cantonale d’éthique de la recherche sur l’être humain, Canton of Vaud, Switzerland; protocol number: 2021–02270; title: Fundamental aspects of object recognition) and in accordance with the Declaration of Helsinki (except for pre-registration). All participants signed informed consent before the experiment and received monetary compensation upon its completion.

### Participants

A total of 18 naive, healthy participants (9 females; age range: 18–23 years old) were recruited for the experiment. Participants had normal or corrected to normal vision, as assessed through the Freiburg acuity test (threshold for inclusion: >1; [73]).

### Apparatus

The stimuli were presented on an ASUS VG248QE LCD monitor with a resolution of 1,920 × 1,080 pixels, a screen size of 24.5 inches, and a refresh rate of 144 Hz. MATLAB R2022b (MathWorks, Natick, MA, USA) along with Psychtoolbox [74] was used to generate the stimuli. During the experiment, participants were seated at a distance of 1.5 m from the screen in a dimly lit room. The stimuli appeared as white with a luminance of 100 cd/m², displayed against a uniform black background with a luminance of 1 cd/m².

### Stimuli and experimental procedure

We used the SQM paradigm, introduced in Otto and colleagues [19], which creates the perception of two motion streams diverging from the center (Fig 1A and 1B). The sequence always began with the presentation of a central vertical line, comprising an upper and a lower segment (the length of each segment is 26.6 arcmin, with a width of 1.2 arcmin) separated by a small vertical gap (2.5 arcmin). Next, pairs of flanking lines appeared progressively further away from the center (horizontal separation between each line is 5 arcmin). The central line and all subsequent pairs of lines were presented for 27.8 ms each, with an interstimulus interval (ISI) of 20.8 ms separating each presentation. The entire stimulus sequence consisted of one central and 5 flanking lines, lasting a total duration of 270.8 ms (Fig 1A).

Participants were instructed to always covertly attend to the right stream. In a randomized order, 6 different conditions were presented (Fig 1C): In the *no vernier* control condition (NV), all lines were straight. In three experimental conditions, one line of the attended stream was offset, with the lower segment shifted either towards the right or the left relative to the upper segment (referred to as a *vernier offset* or V). In these conditions, the vernier offset was presented either in the first central line (V0 condition), in the second flanker line (V2 condition; 100 ms after stimulus onset) or in the fourth flanker line (V4 condition; 200 ms after stimulus onset). In the two last conditions, the central line always had a vernier offset, while another line in the right stream was offset in the opposite direction (referred to as an *anti-vernier* or AV). In these conditions, the opposite vernier was presented either in the second flanker line (V0-AV2 condition) or in the fourth flanker line (V0-AV4 condition). The direction of the vernier offsets was randomly determined before each trial.

In all conditions, participants were asked to indicate whether they perceived a left or right offset along the stream (Fig 1B), even when no vernier was presented. Participants were informed that two opposite verniers could also be presented in the stream and were instructed to report the first one in case they perceived both.

Participants completed a total of 12 blocks, with each block consisting of 96 trials (192 trials per condition, 1,152 trials in total). Before each trial, a central fixation point was presented for 250 ms, followed by an inter-stimulus interval of 750 ms. After the SQM presentation, participants had 3 s to give their response by clicking one of two hand-held buttons. If no response was made within this time, the trial was discarded and repeated at the end of the block. In order to eliminate potential early responses that occurred before the complete presentation of the stimulus sequence, we also excluded trials with reaction times below 300 ms after SQM onset (average number of discarded trials: 8.3 ± 21.2). The inter-trial interval was set at 1 s.

### Offset calibration

Before the experiment proper, the offset sizes (i.e., the horizontal displacement between their upper and lower segments) were determined to achieve comparable performance levels across participants. Streams of straight lines with one single vernier were presented, and a parameter estimation by sequential testing (PEST) procedure [75] was used to adaptively determine offset sizes leading to around 70% to 80% performance. This was performed with a vernier offset separately at the three different spatiotemporal locations tested in the main experiment: the central line (V0), the second flanker line (V2), or the fourth flanker line (V4).

### Behavioral analysis

Performance was determined as the proportion of responses that matched the direction of the first presented vernier offset (Fig 1D). We compared performance between the NV condition and each V or V-AV conditions by means of paired t-tests. The NV condition served as a baseline condition; in this condition performance was determined by comparing the response (left or right reported offset) to a randomly chosen notional offset (mean accuracy was 49% ± 4%). All statistical comparisons were Bonferroni-Holm corrected for multiple comparisons. When the vernier is presented without an anti-vernier, vernier discrimination should be approximately 75%, in line with the calibration performance. Conversely, when an anti-vernier follows the first (central) vernier, the discrimination of this vernier offset should be around 50%, given the integration of the two verniers. Additionally, we anticipated no performance differences related to the specific spatiotemporal locations of the verniers and anti-verniers, as they fell within the same integration window [4]. Behavioral performance aligned with this expectation (see Results section).

Across the six tested conditions, average reaction times ranged from 885 to 920 ms, with no statistically significant difference between conditions (one-way ANOVA, *F*(5, 102) = 0.08, *p* = .99; S1 Data).

### EEG recordings and preprocessing

The EEG data were recorded using a Biosemi Active Two system (Biosemi, Amsterdam, the Netherlands) with a total of 128 Ag–AgCl active electrodes, providing comprehensive scalp coverage. The positioning of the cap was adjusted individually to ensure the Cz electrode was equidistant from the inion and nasion, as well as equidistant from each ear. In addition, the electrooculogram (EOG) was recorded with 4 electrodes positioned 1 cm above and below the right eye and 1 cm lateral to the outer canthi. The recording was referenced to the CMS-DRL ground, maintaining the montage potential close to amplifier zero through a feedback loop. The sampling rate during recording was set at 2,048 Hz.

For EEG preprocessing, EEGLAB was utilized (version v2021.1; [76,77]). The EEG data were first downsampled to 250 Hz. To remove linear trends, detrending was applied [78], followed by a lowpass filter with a cutoff frequency of 40 Hz. The EEG data were then epoched from −1 to 1.5 s relative to the onset of the SQM. To ensure data quality, a visual inspection was conducted to identify and exclude epochs (pre-selected with *pop_jointprob* function) or channels with significant noise or artifacts. Epochs containing eye-related artifacts (e.g., blinks or saccades) overlapping with, or occurring around, the SQM presentation were also removed (but see also Drissi-Daoudi and colleagues [79]) for evidence that integration between two verniers is preserved even when they are presented before and after an eye movement). Then, an ICA decomposition (*pop_runica* function with Picard algorithm; [80,81]) was performed, with a temporary interpolation of the removed channels to maintain a consistent 128-channel configuration across all participants. The ICA decomposition also included fake vertical and horizontal EOG recordings, computed from the 4 EOG channels (differences between the channels located above and below the right eye, and between the channels located on the lateral side of each eye). The visually identified independent components associated with eye or muscular artifacts (pre-selected with *pop_icflag* function) were subsequently removed. Lastly, a final interpolation of the removed channels was conducted, along with an average reference including only the EEG channels.

In total, 6.8% of the electrodes were interpolated, while 3.2% of the epochs and 9.1% of the independent components were removed during the preprocessing procedure.

### EEG analysis

We aimed to investigate whether brain activity reflects unconscious or conscious processing stages by decoding differences in EEG dynamics in two configurations of the SQM: (1) when only a single vernier is presented at different spatiotemporal locations within the stream (V conditions), with all these conditions resulting in the same percept (i.e., one perceived vernier); and (2) when two opposing verniers are presented (V-AV conditions), yet there is no conscious percept of the individual offsets as they integrate.

### EEG decoding

LDA with temporal or cross-condition generalization was used to investigate neural representations related to different SQM conditions or percepts. We implemented LDA with custom-made functions written in MATLAB, based on the recommended settings for EEG data [82,83]. For each participant, LDA with temporal generalization was conducted with a leave-one-pseudo-trial-out cross-validation routine (500 iterations). In each iteration, 80% of the trials were sampled and combined into pseudo-trials (average of 20 trials) for the two classes, i.e., the SQM conditions, that were decoded. The mean of the training set was removed to both testing and training sets, and classifier weights were estimated using a regularized covariance [84–86]. The classifier weights, obtained from the training set for each time point, were applied to predict the classes in the EEG data of the testing set. Performance was evaluated using the AUC. This process was repeated for each time point (cross-temporal decoding, i.e., each classifier is tested on all time points), ranging from −200–1,000 ms, with a sliding window of 7 samples corresponding to a resolution of 70 ms [83]. LDA with cross-condition generalization was performed similarly and with identical parameters, except that the classes used in the training sets differed from those used in the testing sets. For all decoding analyses, EEG data were resampled at 100 Hz and z-scored.

We started by examining V conditions, where the presentation of a single vernier in the stream results in a clear perception of an offset. We used LDA with temporal generalization to discriminate between trials from the control condition (NV) and trials from conditions presenting one single vernier (V0, V2, or V4), irrespective of the behavioral responses (i.e., correct or incorrect report of the vernier offset; Fig 2A and 2B). We also evaluated cross-condition generalization between V conditions. Using classifiers trained with V2 or V4 versus NV conditions, we decoded V0 versus NV conditions (Fig 2C, see also S1 Fig for additional decoding analyses between V conditions).

Second, we investigated the neural representations in the V-AV conditions, where the two opposite verniers integrate with each other. LDA with temporal generalization was used to classify between trials from the NV condition and one of the two conditions presenting two opposite verniers (V0-AV2 or V0-AV4), irrespective of the behavioral responses (i.e., correct or incorrect report of the first vernier offset; Fig 4A; see also Fig 4F for cross-condition generalization between V and V-AV conditions and S2 Fig for cross-condition generalization between V and V-AV conditions, as a function of whether the first or second vernier dominated the reported percept). Next, we conducted additional decoding analyses between V0 and V0-AV2 or V0-AV4 conditions, as well as between the two V-AV conditions (Fig 5).

Lastly, we conducted an LDA analysis with temporal generalization to decode between correct and incorrect reports of the offset within each condition presenting one vernier (V0, V2, and V4; Fig 6). This analysis aimed to specifically identify neural correlates that reflect conscious processing. All parameters remained consistent with those listed above, except for the averaging of trials for pseudo-trials, which was adjusted to 4 due to the reduced number of available trials, especially the incorrect trials (comprising only ~30% of the total for each condition).

### Statistical evaluation of decoding analyses

For all decoding analyses, either with temporal or cross-condition generalization, the statistical evaluation of the 2D matrices of cross-temporal decoding results (participants × time × time) employed cluster-based permutation approaches and surrogate analysis, following established methodologies [86–88]. Clusters were defined as consecutive time points where the decoding successfully exceeded chance levels (chance = 0.5, paired *t* test with *α* = 0.05). The cumulative *t*-values within each cluster were then compared to the maximum sum derived from surrogate clusters (permutations = 10′000). Time points were considered statistically significant if the probability in the surrogate data was <0.05 for the corresponding clusters.

### EEG activation patterns

We estimated activation patterns from the LDA classifiers using the regression-based approach (eq. [8] in [85]). Briefly, this method applies an ordinary least squares regression to derive a pattern that, when multiplied by the discriminant signal obtained via LDA, best approximates the original EEG data used as input to the decoder (electrode × time points × pseudotrials). This approach yields a ‘forward model’ that allows for neurophysiologically interpretable patterns, addressing the known issue of non-interpretable decoder weights [83,85].

We assessed the temporal dynamics of the activation patterns in the context of each decoding analysis (V0, V2, or V4 versus NV, Fig 2; V0-AV2 or V0-AV4 versus NV, Fig 4; V0-AV2 or V0-AV4 versus V0, V0-AV2 versus V0-AV4, Fig 5; correct versus incorrect reports in V0, V2, and V4, Fig 6; see also S1 Fig for V0 versus V2 or V4, and V2 versus V4). For each participant, we extracted the activation patterns over the significant time points.

Across the five initial decoding contrasts (i.e., all V versus NV and all V-AV versus NV conditions; see Figs 2 and 4), we first computed time-by-time dissimilarity matrices based on the spatial topographies of decoder activation in order to characterize the temporal structure of these EEG activation patterns. These activation patterns were then averaged across participants, and the resulting maps were z-scored across electrodes at each time point to control for overall amplitude differences. Next, we calculated pairwise dissimilarities between all time points within the significant decoding window using the Euclidean distance—a standard measure of topographic dissimilarity that quantifies how different two z-scored maps are by summing the squared differences across electrodes.

This procedure yielded a symmetric dissimilarity matrix that reflects the similarity structure of spatial patterns over time. To identify periods during which activation patterns remained relatively stable (i.e., dominated by similar topographies), we transformed this dissimilarity matrix into a graph representation, where each node corresponded to a time point and edges were defined between pairs of time points with dissimilarity values below the median of the entire matrix. This thresholding ensured that only relatively similar patterns were considered connected in the graph.

We then applied the Louvain community detection algorithm [89,90], a widely used method for detecting modular structure in networks, to this graph. In this context, the algorithm grouped together contiguous time points that shared similar activation topographies, thereby identifying clusters of temporally stable EEG patterns. To mitigate potential instabilities in the clustering due to noise at the single-participant level, this procedure was performed on group-averaged data. Nevertheless, we verified the robustness of the main findings across participants by estimating effect sizes (see below). The data-driven clustering allowed us to define distinct temporal clusters characterized by dominant topographic maps (Figs 3A and 4C), which were used in subsequent analyses.

Topographies of the main activation patterns were then generated by averaging over temporal windows corresponding to the identified clusters (Figs 3B and 4D), revealing two highly consistent spatial topographies. To examine their temporal characteristics and contributions to the overall dynamics, we correlated these spatial patterns with the temporal evolution of EEG activation (back-projected time courses; Figs 3C and 4E). For each time course independently, clusters were defined as consecutive time points where the correlation significantly exceeded 0 (paired *t* test with *α* = 0.05). The cumulative t-values within each cluster were then compared to the maximum sum derived from surrogate clusters (permutations = 10′000). Time points were considered statistically significant if the probability in the surrogate data was < 0.05 for the corresponding clusters. Notably, while the topographies derived from dissimilarity matrices were based on group-averaged data, the back-projection plots demonstrate the consistency of these maps and their temporal dynamics across individuals. To further assess these effects at the individual level, we quantified the proportion of participants whose activation patterns showed positive correlations with the dominant topographic maps within the time windows corresponding to the two temporal clusters defined by these maps (Pearson’s *r* > 0 when averaged across each respective time window). It should also be noted that, because the two topographies exhibited largely opposing dipolar scalp distributions, their back-projection onto individual EEG activation patterns resulted in anticorrelated time courses. Interpretation should therefore rely primarily on periods of positive correlation, as these indicate time windows in which the corresponding topography was consistently expressed across participants.

To further assess the reliability of the distinct maps across participants, we estimated Cohen’s *d* (abbreviated as *d* in the Figures) at each electrode and time point by comparing the corresponding activation values across participants against zero (Figs 3A and 4C). The effect sizes here quantify the extent to which activation at a given electrode and time point was consistently different from zero across participants. The presence of large positive and negative effect sizes therefore indicated that the observed topographic patterns were robust and reliably expressed across individuals.

For the additional decoding contrasts (i.e., V0 versus V-AV conditions, V0-AV2 versus V0-AV4 and correct versus incorrect reports in V conditions; see Figs 5 and 6; see also S1 Fig for V0 versus V2 or V4, and V2 versus V4), topographies of the main activation patterns were generated by averaging over the entire significant decoding window (Figs 5B and 6B). In addition, we averaged the two maps identified across all decoding results between V or V-AV versus NV conditions (Figs 3B and 4D) to define two ‘prototypical’ activation patterns associated with the two distinct processing stages (Figs 5C and 6C). We then fitted these prototypes to the individual activation patterns from the additional decoding analyses using separate linear models to assess their relative contributions. For each time course independently, clusters were defined as consecutive time points where the correlation significantly exceeded 0 (paired *t* test with *α* = 0.05). The cumulative *t*-values within each cluster were then compared to the maximum sum derived from surrogate clusters (permutations = 10′000). Time points were considered statistically significant if the probability in the surrogate data was < 0.05 for the corresponding clusters. This analysis confirmed that each decoding result was dominated by a single topography (Figs 5C and 6C). This was further complemented by the estimation of Cohen’s *d* for the difference between each activation pattern and zero at the level of individual electrodes and time points, which highlighted a single, consistent pattern across each significant time window (Figs 5B and 6B).

## Supporting information

S1 Data Behavioral data shown in Fig 1D. (XLSX)

S1 Text Supplementary results. A full description of the results for all supplementary figures.
(DOCX)

S1 Fig LDA between conditions with one vernier. **(A)** Diagonal elements of the temporal generalization matrices (shown in the insets), representing decoding results when training and testing at the same time point (group average AUC and SEM). Significant time windows are highlighted by the horizontal lines at the bottom, showing that classifiers successfully discriminate the conditions (V0 versus V2 or V4, and V2 versus V4; AUC > 0.5, one-tailed cluster-based permutation test, *p* < .05). **(B)** The decoder topographies, averaged across participants, are derived from the averaged activation pattern over the entire significant window of each decoding analysis (left side). These activations patterns were consistently expressed across participants, with many channels and time points showing large effect sizes (difference between each activation pattern and zero, calculated for each electrode and time point |Cohen’s *d*| > 1; right side). **(C)** Prototypical activation patterns (left side) reflecting the occipital (map 1) and parietal topographies (map 2), corresponding to the average of the two distinct activation patterns identified via dissimilarity matrix analysis for all V or V-AV versus NV contrasts (see Figs 3B and 4D), were temporally correlated with the activation patterns found in each decoding window (right side). Gray areas highlight the significant window found in S1A Fig. Blue and red lines represent the occipital and parietal topographies, respectively, and the shaded areas indicate SEM. Significant positive correlations are also highlighted in blue for the occipital topography or in red for the parietal topography (Pearson’s *r* > 0, one-tailed cluster-based permutation test, *p* < .05). The data underlying this Figure can be found in https://doi.org/10.5281/zenodo.20729504.
(TIF)

S2 Fig Temporal and cross-condition generalization as a function of the reported vernier in conditions with two opposite verniers. **(A)** Temporal generalization matrices showing that classifiers successfully discriminate the conditions (V0-AV2 or V0-AV4 versus NV), regardless of the reported vernier (1st or 2nd). Significant clusters are highlighted (AUC > 0.5, one-tailed cluster-based permutation test, *p* < .05). **(B)** Prototypical activation patterns (Figs 5C and 6C), corresponding to the average of the two distinct activation patterns identified via dissimilarity matrix analysis for all V or V-AV versus NV contrasts (Figs 3B and 4D, see Methods), were temporally correlated with the activation patterns found in each decoding window. Gray areas highlight the significant window found in S2A Fig. Blue and red lines represent the first and second topographies, respectively, and the shaded areas indicate SEM. Significant positive correlations are also highlighted in blue for the first map or in red for the second (Pearson’s *r* > 0, one-tailed cluster-based permutation test, *p* < .05). **(C)** Cross-condition generalization matrices showing that classifiers trained with V conditions (V0, V2 or V4) versus NV condition successfully discriminate between the two V-AV conditions (V0-AV2 or V0-AV4 versus NV), regardless of the reported vernier (1st or 2nd). Significant clusters are highlighted (AUC > 0.5, one-tailed cluster-based permutation test, *p* < .05). The data underlying this Figure can be found in https://doi.org/10.5281/zenodo.20729504.
(TIF)

S3 Fig Analysis of ERPs. **(A)** Grand-averaged ERPs across participants and conditions at all electrodes, derived from the EEG signal. Only V0 and NV conditions are shown for illustration purposes. **(B)** Differential ERPs obtained by subtracting the NV condition from each V and V-AV condition. Gray areas indicate the two temporal windows identified in the clustering analysis (Figs 3A and 4C). Blue and red outlines highlight the time windows associated with occipital and parietal contributions to decoding, respectively. **(C)** ERP topographies computed by averaging ERP activity across participants within the two temporal windows identified by clustering analyses. The electrode showing maximal activity within each topography is indicated by a black dot. **(D, E)** ERP time courses at the electrodes showing maximal activity in each time window: occipito-temporal electrode (D) and centro-parietal electrode (E). Only the V0 condition is shown for illustration. Shaded areas indicate SEM. **(F, G)** Differential ERPs at the occipito-temporal (F) and centro-parietal (G) electrodes, computed as each condition minus NV. Colored areas indicate the two temporal windows identified by clustering analyses (Figs 3A and 4C) with blue and red outlines highlighting the time windows associated with occipital and parietal contributions to decoding, respectively. Yellow and red lines represent different V and V-AV conditions, respectively, and the shaded areas indicate SEM. The data underlying this Figure can be found in https://doi.org/10.5281/zenodo.20729504.
(TIF)

## Abbreviations

AUC: area under the curve
EEG: electroencephalography
EOG: electrooculogram
ISI: interstimulus interval
LDA: linear discriminant analysis
NV: no vernier
PEST: parameter estimation by sequential testing
SQM: Sequential Metacontrast
