# Supplementary material for: Sequential neural dynamics underlie unconscious integration and conscious perception of visual stimuli
Source: PLoS Biol. 2026 Jul 6;24(7):e3003894. doi: 10.1371/journal.pbio.3003894 (PMC13362400; doi:10.1371/journal.pbio.3003894)
Supplement: S1 Text — A full description of the results for all supplementary figures. (DOCX) [file pbio.3003894.s002.docx]

## S1 Text. Supplementary results

### Decoding analyses between V conditions

As supplementary analyses, we used linear discriminant analysis (LDA, see Methods) to discriminate between conditions presenting a single vernier (V0, V2, or V4), irrespective of behavioral responses (i.e., whether participants correctly or incorrectly reported the vernier offset). Although these conditions differed physically, because the single vernier appeared at different spatiotemporal locations, they did not differ perceptually (see Otto, 2006; Drissi-Daoudi et al., 2019): in all cases, the offset is integrated into the motion stream and can be reported. The aim was therefore to confirm that the occipital topography, which we associated with unconscious processing of the individual offset, contributed most strongly to decoding performance.

The classifiers (V0 vs. V2 or V4, and V2 vs. V4) successfully discriminated between all pairs of conditions (Area Under the Curve, AUC > 0.5, one-tailed cluster-based permutation test, *p* < .05; S1A Fig). In addition, the onset and duration of significant decoding windows varied systematically depending on the conditions being compared. When V0 was included in the comparison, decoding began around 200 ms, similar to previous analyses comparing V0 vs. NV (Fig 2) and V-AV conditions vs. NV (Fig 4). By contrast, decoding onset was delayed to around 300 ms when comparing V2 and V4, resembling the decoding latencies observed for V2 vs. NV. Finally, when V4 was one of the compared conditions, decoding windows lasted longer, extending beyond 800 ms.

Moreover, as expected, these additional analyses confirmed the dominant contribution of the occipital topography within the significant decoding windows (S1B Fig), indicating that decoding was driven primarily by differences in the topography we associate with unconscious processing. However, we also observed smaller contributions from the parietal topography (S1C Fig). This may reflect the fact that the decoded conditions involved similar processes occurring at temporally shifted time points, as shown by the cross-condition decoding (Fig 2C) and the clustering analysis (Fig 3A) with the V conditions vs. NV. Consequently, the EEG activation patterns underlying decoding may represent a mixture of occipital and parietal topographies across time (S1B Fig). Alternatively, the later dominance of parietal topography observed in comparisons involving V4 (S1C Fig) may indicate that the temporal dynamics of parietal activity vary across conditions, potentially persisting longer in the V4 condition, in line with non-linear temporal dynamics observed across other analyses (see Discussion).

### Temporal and cross-condition generalization as a function of the reported vernier (1^st^ or 2^nd^)

In these supplementary analyses, we decoded the V–AV conditions (V0–AV2 and V0–AV4) from the NV condition, separately for trials in which participants reported the first (i.e., V0) or the second vernier offset (i.e., V2 or V4). The parameters used for LDA with temporal generalization were identical to those used in the analysis comparing correct and incorrect offset reports (see Methods and Fig 6). The aim was to further corroborate two key points: (1) that the occipital topography is time-locked to the onset of the first vernier, even in trials where the second vernier dominates the percept and report—indicating unconscious processing; and (2) that the parietal topography is time-locked to the onset of the perceived (and reported) vernier, as observed in Fig 6B.

The decoder successfully discriminated both V0–AV2 and V0–AV4 trials, regardless of the reported vernier, from NV trials (AUC > 0.5; one-tailed cluster-based permutation test, p < .05; S2A Fig). When participants reported the first vernier, significant decoding emerged around 200 ms, consistent with the analyses including all V–AV trials (vs. NV; Fig 4). In contrast, while similar latencies were found when the second vernier was reported in V0-AV2 condition, the decoding onset was delayed in V0-AV4 condition. At first glance, this pattern might appear inconsistent with the interpretation that the initial decoding and occipital topography are time-locked to the unconsciously processed vernier (which is always V0 in the V–AV conditions).

Because this analysis involved fewer trials due to the split by report, we further assessed the temporal dynamics of the two topographies by correlating the individual activation patterns with the two prototypical topographies identified in the main analyses (Figs 5C and 6C; see Methods). This correlation analysis revealed a significant match with the occipital topography time-locked to the onset of the first vernier and independent of which vernier was reported, followed by the parietal topography, which was delayed and extended in duration when participants reported the second vernier (S2B Fig). Thus, the occipital topography—although still evident in the correlation analysis—may not have survived cluster-based permutation correction in the full temporal generalization matrices.

Together, these findings replicate and extend the main results: the occipital topography is associated with unconscious processing, time-locked to the physical onset of the first vernier (Figs 3C and 4E), whereas the parietal topography reflects the neural correlate of the consciously perceived vernier offset (Fig 6C).

In another supplementary analysis, we decoded V-AV conditions, again separately as a function of the reported vernier, from NV conditions but using classifiers trained on V conditions (V0, V2 or V4 vs. NV). Only V trials where the reported vernier offset was correct were included to ensure that any decoding results could be linked to conscious processing. This approach served two purposes: (1) to confirm that a single vernier offset is consistently represented in V-AV conditions, even when the reported percept does not match the first offset (V0); and (2) to assess whether the temporal decoding delays previously observed in V conditions (see Fig 2B and 2C) also emerge in V-AV conditions, and whether such delays depend systematically on the spatiotemporal location of the reported vernier.

The cross-condition results successfully decoded V-AV conditions from the NV condition in all cases (AUC above 0.5, one-tailed cluster-based permutation test, *p* < .05; S2C Fig). These results confirm that the EEG patterns related to the V-AV and NV conditions differ (see also Fig 4A) and that the neural representation of a vernier is preserved even when two opposite verniers are presented (see also Fig 4F). Moreover, they demonstrate that temporal shifts in decoding between training and testing sets, as indicated by significant clusters that are not aligned along the diagonal, directly depend on whether the first or second vernier is reported (S2C Fig). For example, when classifiers trained on V2 or V4 were tested on the V0-AV4 condition, significant decoding clusters appeared primarily below the diagonal; S2C Fig, lower row), indicating faster decoding in the testing set when the first (central) vernier was reported. This effect was more pronounced for classifiers trained on V4 than on V2. Conversely, when the second vernier was reported in the V0-AV4 condition, the significant decoding cluster appeared above the diagonal with classifiers trained on V2, indicating delayed decoding in the testing set. Lastly, classifiers trained on V4 led to a significant decoding cluster aligned along the diagonal, confirming the absence of temporal shift.

Together, these results confirm that the neural processes underlying V–AV conditions—regardless of which vernier is reported—closely resemble those observed in V conditions, following a similar sequence of unconscious and conscious processing stages (see Figs 2 and 3 for comparison). Moreover, consistent with the results shown in S1 Fig, they indicate that the temporal dynamics of these processes, particularly those associated with the parietal topography, vary as a function of the perceptual outcome—that is, depending on which vernier offset dominates the final percept and behavioral report.

### Event-related potential analysis

Our decoding analyses showed that decoding performance was driven either by the presence of two successive distinct maps—namely, the occipital and parietal topography (for V or V-AV conditions vs. NV conditions; see Figs 3 and 4)—or by the presence of a single dominant map. The occipital topography contributed most strongly when unconscious processing differed despite a similar integrated percept (e.g., between V and V-AV conditions, or among V and V-AV conditions; see Fig 5 and S1 Fig), whereas the parietal topography dominated when perceptual reports differed (i.e., correct vs. incorrect reports in V conditions; see Fig 6).

In the Discussion, these two distinct topographies are compared with well-known event-related potential (ERP) components. In particular, we relate the occipital topography to the N170/VAN component and the parietal topography to the classic P300. In these additional plots, we further validate the involvement of these specific ERP components.

Condition-specific ERPs were first computed for each participant by averaging EEG epochs across trials within each condition. A pre-stimulus baseline correction was applied using the interval from −450 to −50 ms relative to stimulus onset. This resulted in a channel × time ERP matrix for each participant and condition. Overall, inspection of the grand-averaged ERPs revealed highly similar patterns across conditions (examples are shown for V0 and NV conditions in S3A Fig).

To isolate condition-specific activity, differential ERPs were computed by subtracting the NV condition from each experimental condition (S3B Fig). Difference maps were then normalized by the across-subject standard deviation to estimate effect sizes. The results revealed strongest effects at different latencies depending on condition, with differences emerging later in V2 and V4, consistent with the decoding onset reported in Fig 2B. Moreover, the strongest effects aligned with the two temporal windows identified by the cluster analyses (Figs 3A and 4C), and the corresponding topographies matched the EEG patterns contributing to decoding within these windows (example shown for V0 in S3C Fig; see Figs 3B and 4D for comparison). In both windows, we identified the electrode showing maximal effect. This corresponded to an occipito-temporal electrode in the first window and a centro-parietal electrode in the second window (S3C Fig). Because highly similar electrodes were selected across all conditions, we used these two electrodes as the basis for subsequent electrode-specific ERP plots for each condition.

For both the occipito-temporal and centro-parietal electrode, we estimated the raw ERPs (example shown for V0 in S3D and S3E Fig) and differential ERPs (V or V-AV conditions minus NV condition; S3F and S3G Fig). At the group level, V and V-AV conditions showed a clear increase in negative amplitude relative to the NV condition at the occipito-temporal electrode (S3F Fig), starting around 200 ms for V0 and V-AV conditions, and emerging later for V2 and V4 conditions. For each condition, this effect closely aligned with the onset of significant decoding (vs. NV condition) and with the time window reflecting a contribution of the occipital topography to decoding (Figs 3C and 4E). These findings therefore support our interpretation that differences between V or V-AV conditions and the NV condition arise from modulation of early occipital activity, potentially consistent with a stronger N170/VAN-like response, time-locked to the onset of the first vernier in the stream. At the centro-parietal electrode, a later positive modulation distinguished V and V-AV conditions from the NV condition (S3G Fig), with the earliest onset starting around 360 ms but varying again across conditions with later presentation of a single vernier. This pattern closely matched the second time window identified in the decoding analyses and associated with the contribution of the parietal topography (Figs 3C and 4E) and is therefore consistent with the interpretation that the later effects reflect a P300-like response.
