# Supplementary figures and images for: Sequential neural dynamics underlie unconscious integration and conscious perception of visual stimuli
Source: PLoS Biol. 2026 Jul 6;24(7):e3003894. doi: 10.1371/journal.pbio.3003894 (PMC13362400; doi:10.1371/journal.pbio.3003894)

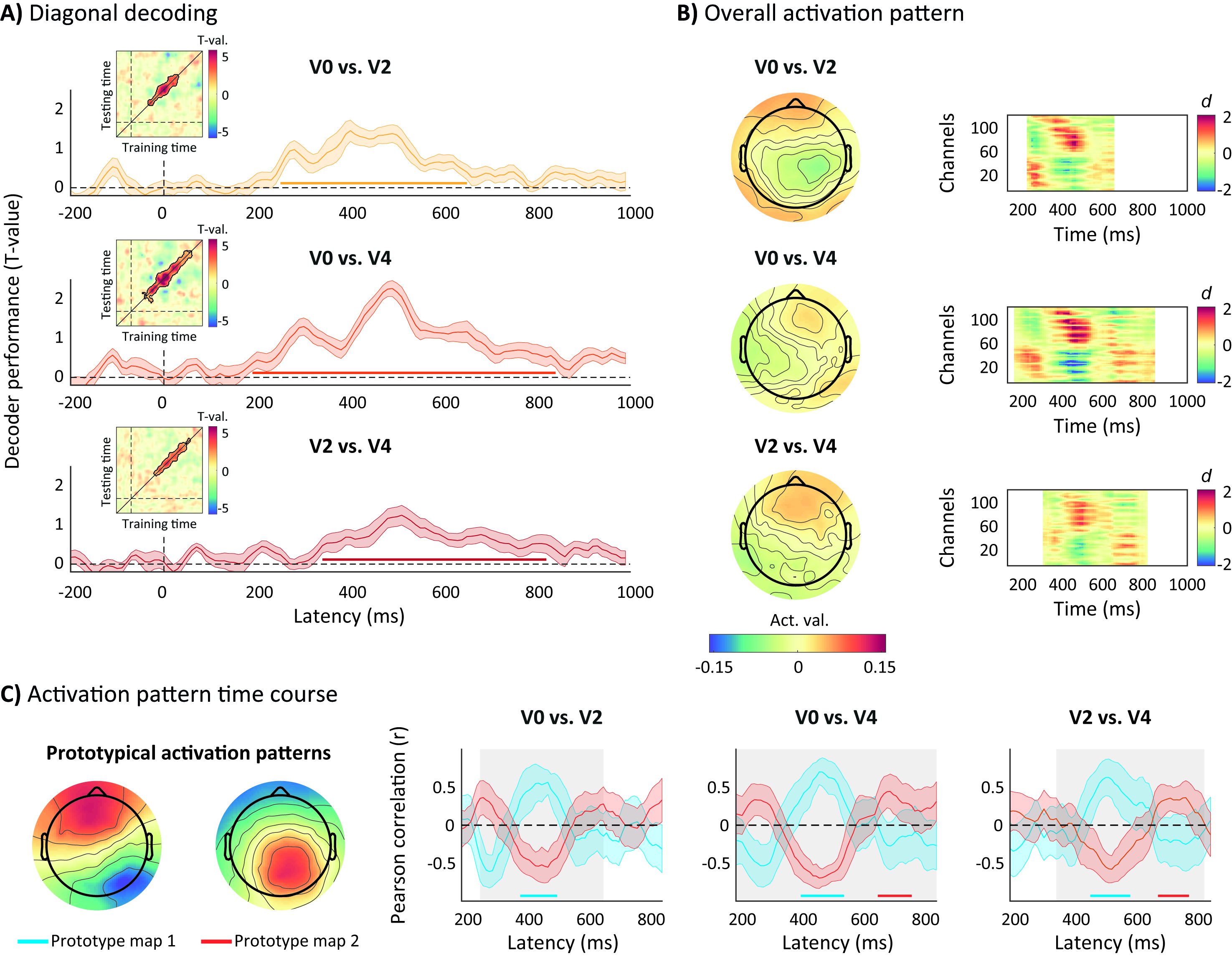

Supplement: S1 Fig — (A) Diagonal elements of the temporal generalization matrices (shown in the insets), representing decoding results when training and testing at the same time point (group average AUC and SEM). Significant time windows are highlighted by the horizontal lines at the bottom, showing that classifiers successfully discriminate the conditions (V0 versus V2 or V4, and V2 versus V4; AUC > 0.5, one-tailed cluster-based permutation test, p < .05). (B) The decoder topographies, averaged across participants, are derived from the averaged activation pattern over the entire significant window of each decoding analysis (left side). These activations patterns were consistently expressed across participants, with many channels and time points showing large effect sizes (difference between each activation pattern and zero, calculated for each electrode and time point |Cohen’s d| > 1; right side). (C) Prototypical activation patterns (left side) reflecting the occipital (map 1) and parietal topographies (map 2), corresponding to the average of the two distinct activation patterns identified via dissimilarity matrix analysis for all V or V-AV versus NV contrasts (see Figs 3B and 4D), were temporally correlated with the activation patterns found in each decoding window (right side). Gray areas highlight the significant window found in S1A Fig. Blue and red lines represent the occipital and parietal topographies, respectively, and the shaded areas indicate SEM. Significant positive correlations are also highlighted in blue for the occipital topography or in red for the parietal topography (Pearson’s r > 0, one-tailed cluster-based permutation test, p < .05). The data underlying this Figure can be found in https://doi.org/10.5281/zenodo.20729504. (TIF) [file pbio.3003894.s003.tif]

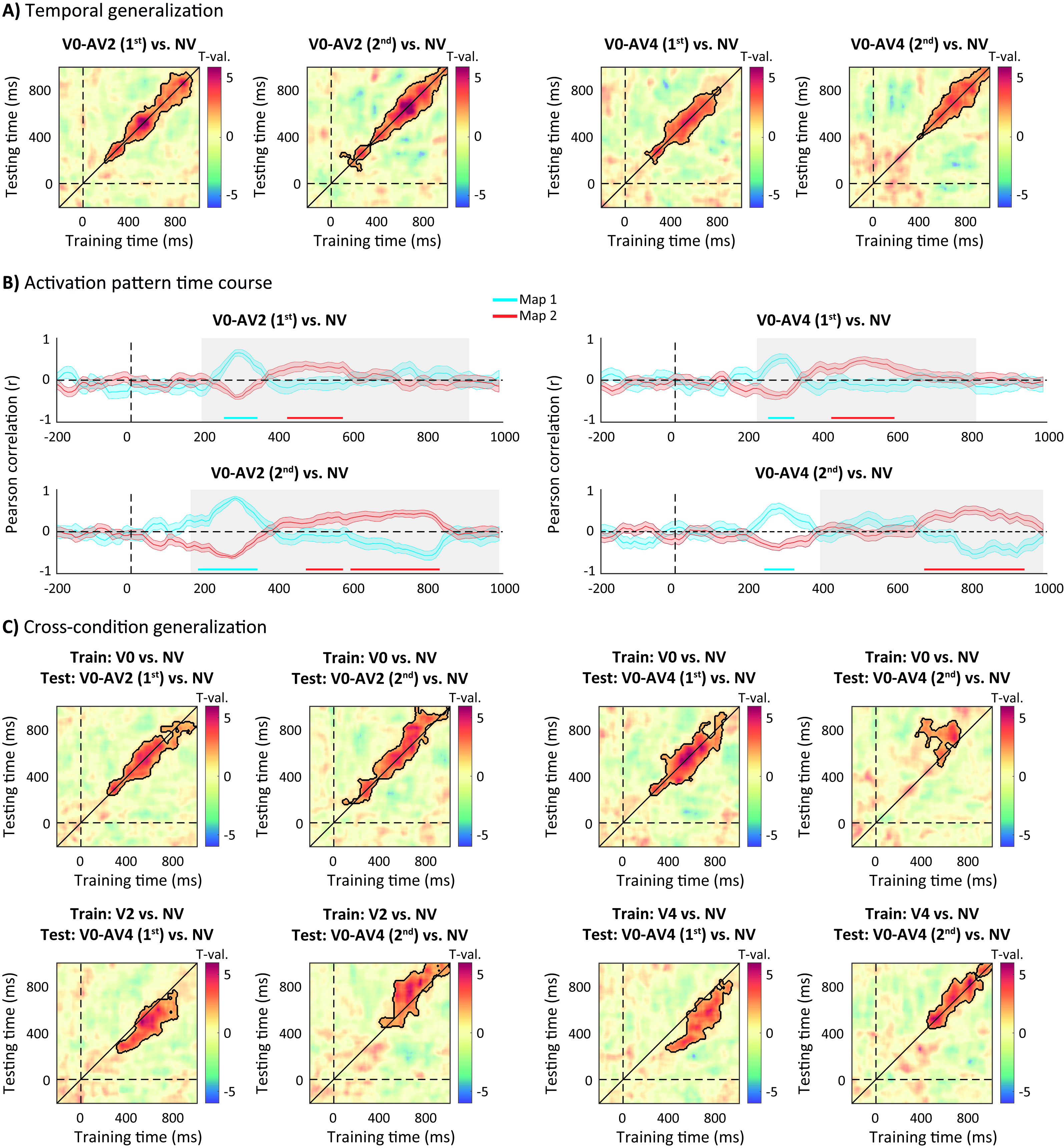

Supplement: S2 Fig — (A) Temporal generalization matrices showing that classifiers successfully discriminate the conditions (V0-AV2 or V0-AV4 versus NV), regardless of the reported vernier (1st or 2nd). Significant clusters are highlighted (AUC > 0.5, one-tailed cluster-based permutation test, p < .05). (B) Prototypical activation patterns (Figs 5C and 6C), corresponding to the average of the two distinct activation patterns identified via dissimilarity matrix analysis for all V or V-AV versus NV contrasts (Figs 3B and 4D, see Methods), were temporally correlated with the activation patterns found in each decoding window. Gray areas highlight the significant window found in S2A Fig. Blue and red lines represent the first and second topographies, respectively, and the shaded areas indicate SEM. Significant positive correlations are also highlighted in blue for the first map or in red for the second (Pearson’s r > 0, one-tailed cluster-based permutation test, p < .05). (C) Cross-condition generalization matrices showing that classifiers trained with V conditions (V0, V2 or V4) versus NV condition successfully discriminate between the two V-AV conditions (V0-AV2 or V0-AV4 versus NV), regardless of the reported vernier (1st or 2nd). Significant clusters are highlighted (AUC > 0.5, one-tailed cluster-based permutation test, p < .05). The data underlying this Figure can be found in https://doi.org/10.5281/zenodo.20729504. (TIF) [file pbio.3003894.s004.tif]

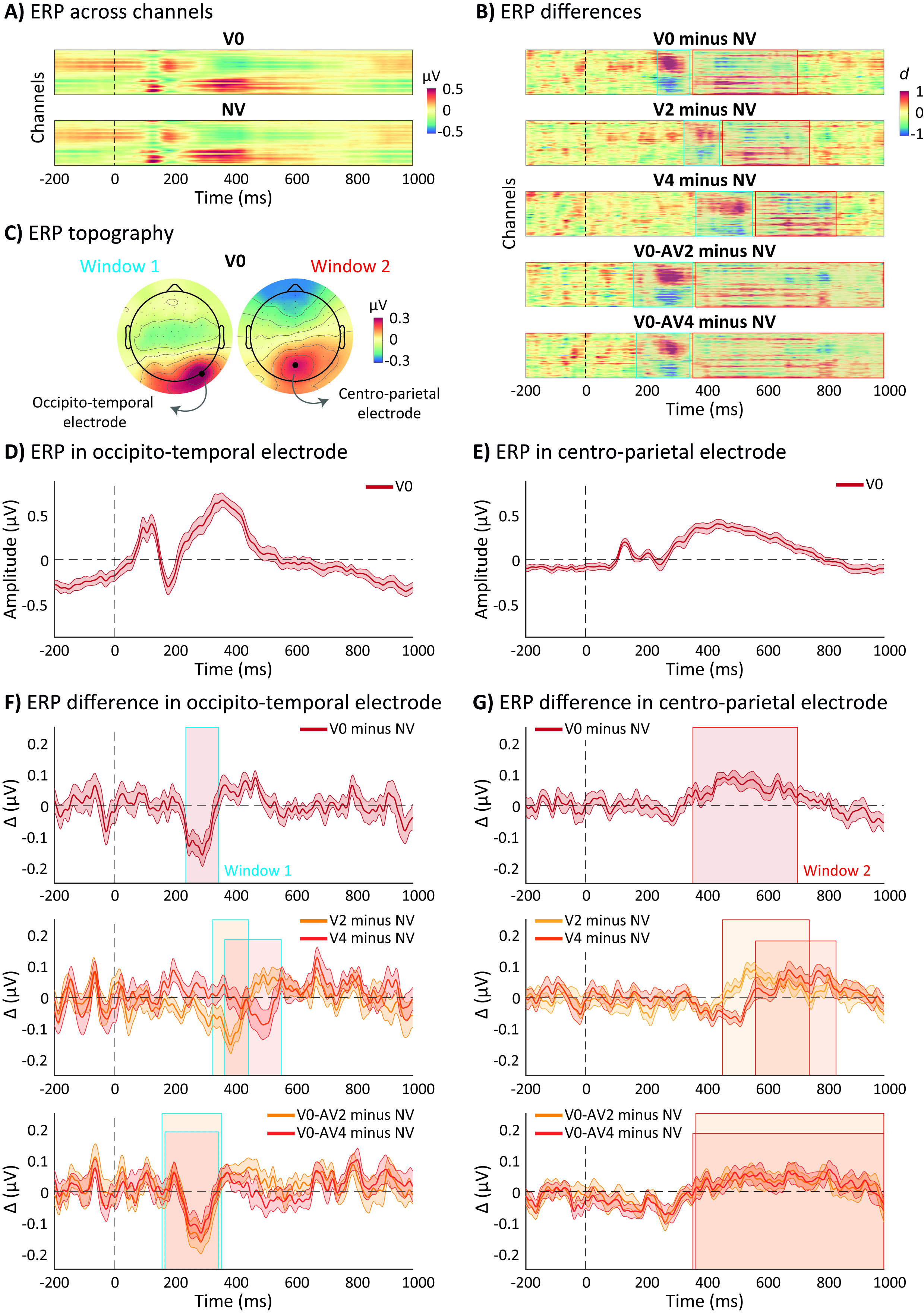

Supplement: S3 Fig — (A) Grand-averaged ERPs across participants and conditions at all electrodes, derived from the EEG signal. Only V0 and NV conditions are shown for illustration purposes. (B) Differential ERPs obtained by subtracting the NV condition from each V and V-AV condition. Gray areas indicate the two temporal windows identified in the clustering analysis (Figs 3A and 4C). Blue and red outlines highlight the time windows associated with occipital and parietal contributions to decoding, respectively. (C) ERP topographies computed by averaging ERP activity across participants within the two temporal windows identified by clustering analyses. The electrode showing maximal activity within each topography is indicated by a black dot. (D, E) ERP time courses at the electrodes showing maximal activity in each time window: occipito-temporal electrode (D) and centro-parietal electrode (E). Only the V0 condition is shown for illustration. Shaded areas indicate SEM. (F, G) Differential ERPs at the occipito-temporal (F) and centro-parietal (G) electrodes, computed as each condition minus NV. Colored areas indicate the two temporal windows identified by clustering analyses (Figs 3A and 4C) with blue and red outlines highlighting the time windows associated with occipital and parietal contributions to decoding, respectively. Yellow and red lines represent different V and V-AV conditions, respectively, and the shaded areas indicate SEM. The data underlying this Figure can be found in https://doi.org/10.5281/zenodo.20729504. (TIF) [file pbio.3003894.s005.tif]
