# Supplementary material for: Sequential neural dynamics underlie unconscious integration and conscious perception of visual stimuli
Source: PLoS Biol. 2026 Jul 6;24(7):e3003894. doi: 10.1371/journal.pbio.3003894 (PMC13362400; doi:10.1371/journal.pbio.3003894)
Supplement: Attachment — Submitted filename: PlosBiol_Reply_R1.docx [file pbio.3003894.s008.docx]

We would like to thank the Editor and the Reviewers for their positive evaluations of our study and their valuable suggestions. We hope we have carefully addressed all the Reviewers' concerns in the point-by-point response below, with corresponding changes made to the manuscript. These changes are highlighted in red in the main text. For clarity, we have organized the Reviewers' comments and our responses in a bullet-point list.

We hope that the Editor and Reviewers will now find our manuscript suitable for publication.

# REVIEWS:

## Reviewer #1

Menétrey et al. "Neural evidence for a two-stage model of visual processing"

Comments
The paper reports the results of an EEG decoding analysis of the interesting, as well as slightly vexing, SQM paradigm. The study has been expertly conducted, and the results are rather beautiful. The conclusions drawn also seem clear and appropriate, and as such I have little to complain overall. Nevertheless, I still have a few specific points/queries:

We very much thank the Reviewer for the positive assessment of our manuscript and helpful references.

1. Decoding in both the V (0, 2, and 4) the V-AV (2 and 4) conditions showed that the presence of the (last) Vernier element was delayed as its onset occurred later in the stream. This is a nice result, but I noticed the delay is not constant. Between V0 and V2, there is about 90 ms, close to the actual 100 ms SOA difference, but between 2 and 4, the delay is only 40 ms. What could be the reason for this? Might it be some kind of perceptual acceleration, caused by the succession of stimulus displays, like we found in the missing element task (Wolff & Akyurek, in press)? I'd be interested in the authors' take on this.

   Indeed, the delays are different, as noted in the Discussion (lines 432-435). In line with the Reviewer’s suggestion, we have now added the sentence: “One possibility is that the occurrence of a vernier offset initiates an unconscious processing window, while subsequent offsets or the termination of the SQM stream may accelerate the collapse of the sequence into a unified conscious percept (Wolff & Akyürek, 2026).”
2. Scholte et al. have recently claimed that binding (=integration?) does not exist in the brain, or is at least not needed conceptually. I thought it may be interesting to consider this idea in the light of the present results, which to me at least seem to suggest the opposite. I do apologize in advance if this only leads to a garden path. <https://doi.org/10.1016/j.tics.2025.03.002>

   This is an interesting point. To our understanding, Scholte critiques the classical view according to which features such as color, motion, and orientation are initially processed in parallel by separate modules and therefore require a subsequent “binding” stage, for example through focused attention that selectively combines features at specific spatial locations (as in Treisman’s Feature Integration Theory, FIT). Instead, he argues that the brain represents naturally co-occurring patterns of information and spatiotemporal structure, without the need to bind isolated features.

   What we show in the SQM is that features distributed across different spatial and temporal locations can be integrated unconsciously over several hundred milliseconds. This integration does not necessarily imply separate modules that are later combined. Rather, it depends on how the brain interprets the spatiotemporal structure of the stimulus and assigns individual events, initially processed unconsciously, to a common perceptual object or trajectory. Whether this requires an explicit “binding” stage (as in Scholte’s criticized definition), or is better understood as perception operating over structured spatiotemporal information rather than isolated features, remains an open question. Thus, our results do not provide direct evidence to adjudicate between these theoretical views.

We believe this is an interesting but speculative issue that is beyond the scope of the present study.

3. In the discussion, reference is made to the N170/VAN component of the ERP. It might be worthwhile to compute and actually show this ERP from the present data (time-locked to the Vernier), rather than only the decoding scores and their topography, to further substantiate this comparison.

   We now include plots of the ERPs related to our main findings as Supplementary Figure 4 (lines 1212-1278).

Signed,
Elkan Akyürek

## Reviewer #2

(Max Levinson also signed his report): The manuscript "Neural evidence for a two-stage model of visual processing" by Menétrey et al. is an elegant demonstration of the two-stage model for unconscious/conscious processing espoused by Herzog and colleagues over the past decade. The results are striking and nicely contextualized within this framework. They find that neural activity during the sequential metacontrast task (SQM) consists of two temporally-distinct stages: one dominated by occipital activity corresponding to unconscious processing of vernier offsets, followed by a parietal topography corresponding to conscious perception. The paper has clear implications for the broader fields of perception and consciousness.

Although the findings largely support the two-stage model, the manuscript is written as if this model was already established fact. The authors should soften these claims, especially as the purpose of this study is to discover two neural stages that would support the theoretical model. Similarly, claims about the phenomenology of SQM are made without behavioral evidence, such as that participants never perceive two offsets in V-AV conditions. Some more caution throughout the manuscript would improve its readability and, I think, better demonstrate the novelty of these interesting and likely very impactful findings.

We thank the Reviewer for the thorough evaluation of our work. We have made several changes to the manuscript in accordance with the Reviewer’s comments.

Specific comments follow below.

Abstract

1. The first sentence already begs the question of the two-stage model hypothesis. Postdictive effects do not necessarily require periods of unconscious processing if, for example, consciousness is continuously updated and we simply forget previous experiences.

   In the SQM, earlier events are not simply forgotten but are systematically influenced by later ones. Previous studies (see lines 50-67) showed that when the stream contains two verniers with opposite offsets, their offsets are integrated and performance is at chance. If the first vernier were forgotten, observers would report the offset of the second, leading to systematically below-chance performance when accuracy is defined relative to the first offset.
2. Line 33: Similarly, my understanding is that the purpose of this experiment is to use EEG to discover whether there are two subsequent stages that correspond to unconscious and conscious processing. But here it is already claimed that the stages are "well separated in time" in the SQM, presumably concluded from prior behavioral data. However, if our conscious experience is of an integrated offset from the beginning until the end of the trial, then conscious and unconscious processing appear to fully overlap in time.

   In this work, we aimed to identify EEG markers reflecting the two stages previously suggested by behavioral studies. Moreover, postdictive effects suggest that the integrated conscious percept is necessarily delayed relative to the physical stimuli. In the example of the V–AV condition, the conscious percept can only arise after the presentation of the second offset; otherwise, the two offsets would be perceived as individual events. Thus, unconscious and conscious processing cannot fully overlap in time. We clarified this point in the Abstract (lines 27-32).

Introduction

3. Lines 54-55: This sentence was a bit confusing because "lines" is supposed to refer to the vertical pairs of lines at each stimulus increment, but I initially read it as referring to each individual line segment. (Each individual line is always vertically straight). Maybe using a term like "line pair" would be helpful.

   We clarified the sentence (lines 53-54): “When one of the lines contains a horizontal vernier offset (i.e., the lower segment of the line is slightly shifted leftward or rightward relative to the upper segment), all the other lines are perceived as having the same offset, even though they are actually straight.”
4. Line 65: I suggest explicitly addressing the potential role of motion for producing such long windows in the SQM. Non-moving visual stimuli might be integrated over shorter windows.

   Previous results have shown that long-lasting integration can occur without motion perception and with static stimuli (see Scharnowski et al., 2009 and Pilz et al., 2013; lines 409-419). However, it remains possible that the integration window may be shorter for different stimuli compared to dynamic streams such as in the SQM. We now mention this point (lines 420-423): “Although the unconscious integration window appears relatively long in the SQM, consistent with the fact that individual vernier offsets are not perceived as separate events, its duration may also reflect the demands of motion processing, whereas in other tasks shorter integration windows may allow conscious perception to emerge earlier.”
5. Lines 87-90: unclear if this "rationale" is a hypothesis about how the SQM works, or findings from previous behavioral studies (if so, they should be cited).

   Thank you very much. The rationale refers to previous findings (see lines 50-67). We added the reference where we layed out the rationale (Herzog et al., 2020; line 91) and provides the basis for the two-stage model proposal.
6. Fig. 1D: I recommend removing the colored backgrounds in the bar plots - they are unnecessary and make the individual dots hard to see.

   True. We removed the colored backgrounds and added colored arrows to indicate dominance levels (see Figure 1D).
7. Fig. 1D legend: here it is claimed that V-AV performance is at 50% because participants could not perceive the individual offsets, but later in the paper it is claimed that perception is biased towards one or the other offset in each individual trial.

   We completed the legend as follows (lines 82-84): “In V-AV conditions, participants could not perceive the individual vernier offsets, and hence performance is at 50%. This indicates that both vernier offsets contribute equally to the integrated percept, or that each offset is reported with equal probability in a given trial.”
   In addition, we now explicitly acknowledge in the Discussion (lines 484-497) that the use of a 2-AFC method constitutes a limitation of the current study, as it does not provide direct evidence about what is actually perceived in the V–AV condition (e.g., a single offset versus a straight line): “While many previous findings support the long-lasting unconscious integration of visual features in the SQM (Otto, 2006; Otto et al., 2010; Plomp et al., 2009; Drissi-Daoudi et al., 2019; Vogelsang et al., 2023, 2024), the typical use of a forced-choice task based on the offset direction of the first vernier, as employed here, does not allow one to directly infer the exact percept experienced by the participants in the V–AV condition. That is, one cannot infer whether participants perceive a perfectly straight line or a weaker residual offset resulting from non-uniform integration. A recent study showed that the reported offset direction in V–AV conditions, whether consistent with the first or the second vernier, can be predicted by pre-stimulus EEG fluctuations in the alpha band (Menétrey et al., 2023). This finding suggests that integration may indeed vary across trials, with fluctuations in the relative dominance of the first or second vernier. Importantly, regardless of whether integration is uniform or non-uniform, our results show that the spatiotemporal location of each individual vernier, although not consciously accessible as a separate event because all elements are integrated within the SQM stream, can nevertheless be decoded from a specific occipital topography."

Results

8. Line 160: A large mean effect size does not necessarily imply consistency across participants. Better would be to report the prevalence of the effect in the sample, such as the percentage of participants showing the effect in the same direction.

   We agree that a large mean effect size alone does not necessarily imply consistency across participants. At the same time, reporting only the percentage of participants showing an effect in the same direction may also be misleading, as it does not take into account the effect magnitude or measurement noise. Nevertheless, following the Reviewer’s suggestion, we now report this metric (see lines 165-171, 205-209 and 737-745), which indicates a highly consistent pattern across participants.
9. Lines 161-163: Negative values in the first topography and positive values in the second topography are both labeled as "stronger activation" or "increased activation."

   We thank the Reviewer for noting this point. When projecting the classifier weights onto EEG sensor space, darker colors (red or blue) indicate channels that contribute most strongly to decoding. We clarified this in the relevant passage (lines 157-161), as well as in other instances throughout the manuscript (e.g., line 260) by referring to “contributions to decoding” rather than “activation”.
10. Fig. 3A, bottom: Cohen's d is labeled as d' in many figures.

    We now use d (instead of d′) as an abbreviation in the figures for visualization and space considerations, but we retain Cohen’s d in the main text. This is now specified in the Methods section (lines 746-748): “To further assess the reliability of the distinct maps across participants, we estimated Cohen’s *d* (abbreviated as d in the figures)”.
11. Fig. 3C: is it relevant that the two topographies are anti-correlated? (as opposed to one map's r staying at 0 while the other significantly increases)

This stems from the fact that the two topographies have largely opposite polarities. As a result, when one pattern is present, the other shows a corresponding inverted (negative) pattern across the same spatial layout. We clarify this and the way to interpret it in the revised Methods section (lines 740-745): “It should also be noted that, because the two topographies exhibited largely opposing dipolar scalp distributions, their back-projection onto individual EEG activation patterns resulted in anticorrelated time courses. Interpretation should therefore rely primarily on periods of positive correlation, as these indicate time windows in which the corresponding topography was consistently expressed across participants.”. In line with this interpretation, we have removed statistical tests based on negative values relative to zero, as well as direct comparisons between the two reconstructed topography time courses, as these do not provide independent evidence beyond the positive-expression periods (Figures 3C, 4E, 5C, and 6C).

12. Line 175: I am not sure what the "difference between each activation pattern and zero" means. Is it the average activation in each electrode, or some measure of the topographical pattern?

By “difference between each activation pattern and zero,” we refer to a univariate test at each electrode and time point, where the activation values (across participants) are compared against zero (i.e., a null value). Specifically, Cohen’s d quantifies the standardized mean activation relative to zero at each electrode/time point. Thus, it is not an average topographical pattern measure, but rather an effect size indicating whether the activation at each electrode and time point is reliably different from zero across participants. We clarify this in the revised manuscript (lines 746-752, see also figure legend, e.g. lines 176-179): “To further assess the reliability of the distinct maps across participants, we estimated Cohen’s d (abbreviated as d in the figures) at each electrode and time point by comparing the corresponding activation values across participants against zero (Figures 3A and 4C). The effect sizes here quantify the extent to which activation at a given electrode and time point was consistently different from zero across participants. The presence of large positive and negative effect sizes therefore indicated that the observed topographic patterns were robust and reliably expressed across individuals.”

13. Line 186: "Location" is unclear, as it could refer to the increment in the SQM sequence (temporal location), the horizontal position of the offset, or the left versus right streams. From the context of the sentence I believe it is referring to the temporal location. If so, what is the evidence that the temporal location was not consciously perceived?

    We added the term “spatiotemporal” before each instance of “location” to clarify that we refer to both the spatial and temporal position of the vernier in the stream (e.g., lines 106, 113, 125...).
14. Furthermore, none of the results so far show decoding of the temporal location of the vernier (e.g., V2 vs V4). There is only decoding of one temporal location versus no vernier (e.g. V2 vs NV).

    We agree and have now included these analyses in the revised manuscript. As we considered the cross-condition decoding results already informative regarding the temporal location of the verniers, and for reasons of space, these additional analyses are reported in the Supplementary Materials (lines 1060-1091, see Supplementary Figure 1) but are discussed in the main text (lines 260-264): "We further corroborated the dominant occipital contribution by decoding between V conditions involving physically different sequences of events that nevertheless led to the common conscious perception of a single integrated offset (V0 vs. V2 or V4, and V2 vs. V4). These results further suggest that this topography reflects the processing of the spatiotemporal location of a vernier offset prior to its integration within the SQM stream (see Supplementary Figure 1)."
15. Line 199: This result does not appear related to the weak integration hypothesis from the previous sentence. One would expect to decode V-AV from NV trials even if the two offsets are perfectly integrated unconsciously (so no offset is perceived).

We clarified the whole section in the revised manuscript (see lines 187-296, see also response to point 16).

16. Line 207: In general, the article is missing more a convincing argument that the two offsets are always mandatorily integrated in conscious perception. This claim is made several times, sometimes with citations to previous work, but it is not tested in the present study (the participants are not asked whether they perceive one or two offsets). It would be helpful to add some more explanation for how the cited studies show that the two offsets are always integrated into one.

    When a single vernier is presented (V condition), participants can easily report its offset. However, participants can no longer reliably report the same vernier offset when an anti-vernier is added in the stream (V–AV condition). In this case, performance drops to chance level. These findings have been interpreted as evidence for mandatory integration. Moreover, previous studies (Driss-Daoudi, 2019; Vogelsang et al., 2023) demonstrated that integration is not simply determined by spatiotemporal proximity, but occurs only when offsets are presented within a discrete temporal window (approximately 400 ms). When two verniers are separated by more than 400 ms, participants are able to perceive and report both offsets independently (see lines 50-67).

Yet, the Reviewer is correct and indeed, we rely on an established paradigm without testing, explicitly, what participants perceive in the V-AV condition. This is a limitation of the forced-choice task we now recognize in the revised Discussion (lines 484-497 and response to point 7).

As an additional note, we would like to mention here that we have recently conducted a follow-up behavioral study using an adjustment method in which participants directly reported their percept by drawing the perceived vernier (e.g., a straight line or an offset with varying amplitude), as well as indicating the number of offsets perceived in the stream. The results of this new study clearly show that participants perceive and report a single offset in V–AV trials. The results were presented at ECVP 2025 (Holdsworth, M., Menétrey, M. Q., Oluk, C., and Herzog, M. H. (2025). A continuous adjustment task allows for better understanding of visual feature integration in a Sequential Metacontrast Paradigm. 77th European Conference on Visual Perception, Mainz, Germany). The manuscript is currently in preparation, and unfortunately there is for the moment no reference that we can cite in this manuscript.

17. In addition, if the two offsets are not perfectly integrated and subjects still perceive a slight offset, V-AV versus NV decoding could pick up differences in conscious processing but not unconscious processing.

    We agree. However, as in the V conditions, any residual offset in V–AV trials would be expected to propagate along the entire stream and contribute to the integrated percept. Therefore, decoding results that specifically track the spatiotemporal location of the second vernier (e.g., lines 238-250) are unlikely to be explained by conscious processing alone.
18. Lines 248 and 253: same concern that subjects were not asked if they perceived a second offset or not.

    We have clarified these sentences (see response to point 15). The general rationale is that the second vernier is necessarily integrated with the first vernier (and therefore not consciously perceived as an independent event), as participants are not able to report the offset direction of the first vernier in V-AV conditions (which they are instructed to do; we have clarified this point in the Methods section as well, see lines 553-555: "Participants were informed that two opposite verniers could also be presented in the stream and were instructed to report the first one in case they perceived both."). We also want to highlight that Drissi-Daoudi et al. (2019) provide additional evidence showing that integration in the V–AV condition is unaffected by whether participants are instructed to report the first or the second vernier.
19. Line 251: V0-AV2 and V0-AV4 may weigh the two offsets differently and produce different conscious experiences of the integrated offset. Fig. 1D suggests this may be the case. The decoder could be picking up the difference in conscious perception, not the temporal position of the second vernier.

    We are not able to specifically test whether the percept differs between these two V–AV conditions, as we now discuss (see response to points 7 and 16). The only indication we have is that performance does not differ at the behavioral level, suggesting that the integration of the two verniers is similar across conditions.

    However, we can further address the Reviewer’s concern based on the EEG results. Specifically, decoding analyses between V–AV trials and NV trials, split according to the reported offset (i.e., whether the first or the second vernier was reported), reveal the same two topographies observed in the V or V–AV vs. NV comparisons (see Supplementary Material, lines 1131-1193). Importantly, these analyses also show that the first topography emerges at a comparable latency regardless of the relative weighting of the two offsets, whereas the second topography varies as a function of the reported offset.

Taken together, these findings suggest that the occipital topography is unlikely to reflect differences in conscious percepts between V–AV2 and V–AV4, whereas the parietal topography may indeed be more sensitive to differences in the final reported percept.

20. Line 256: I suggest labeling the first topography as "occipital" and the second as "parietal", or something like that, to help orient the reader when discussing the two topographies.

    Following the Reviewer’s suggestion, we now refer to the “first” and “second” topographies as the “occipital” and “parietal” topographies, respectively (see lines 157-161).
21. Lines 267 and 321: There is no temporal generalization in figures 5 and 6.

    We now present all the temporal generalization matrices in Supplementary Figure 2 (see also lines 1111-1124).
22. Line 297: How were the offset directions and button mappings randomized / counterbalanced? Is there any risk that the correct v. incorrect decoder is picking up either unconscious offset direction or motor signals?

    We thank the Reviewer for noting that this information was missing. As now specified in lines 550-551, “the direction of the vernier offsets was randomly determined before each trial.” This randomization ensured that offset direction was not systematically associated with a specific response, thereby minimizing the risk that the decoder captured motor-related signals.
23. I imagine an offset might be missed if the eyes are moving during its brief presentation (27.8 ms), and eye movement could therefore account for correct vs. incorrect decoding. Participants were instructed to covertly attend but, to my understanding, eye position was not monitored. It would be informative to check if the EOG data differentiate correct from incorrect trials, particularly during 1) the offset presentation, 2) the periods of significant correct vs. incorrect decoding.

    Eye movement artifacts (blinks and saccades) were visually inspected, and epochs containing eye activity overlapping with, or occurring around, the time of SQM presentation were excluded from the analyses. In addition, EOG channels were used to improve the identification and isolation of residual ocular components via ICA, which were subsequently removed from the EEG data. We therefore do not consider eye movements to be a plausible confound in the reported effects. A previous study also showed that when an eye movement was made during SQM presentation (Drissi-Daoudi et al., 2020), offsets presented before and after the saccade were still integrated. We now report these two points in lines 608-611: “Epochs containing eye-related artifacts (e.g., blinks or saccades) overlapping with, or occurring around, the SQM presentation were also removed (but see also Drissi-Daoudi et al., 2020 for evidence that integration between two verniers is preserved even when they are presented before and after an eye movement).”
    Importantly, the time window of significant correct versus incorrect decoding occurred well after the end of the SQM sequence. Moreover, the corresponding topographies do not show frontal distributions typically associated with ocular artifacts. Together, these observations further indicate that eye movements are unlikely to account for the reported decoding effects.

Materials and Methods

24. Why was 40 Hz chosen for the lowpass? This precludes detecting gamma-range activity that is often considered a medium for unconscious visual processing.

    In our analysis, we focused on ERP-based decoding and applied a 40 Hz low-pass filter primarily to reduce high-frequency noise and improve signal-to-noise ratio. This choice is also consistent with common practice in ERP decoding studies, where even more conservative cut-offs are sometimes used (e.g., Bae & Luck, 2019, Psychological Science, who used a 6 Hz low-pass filter). Nevertheless, we recognize that an interesting future step would be to assess the contribution of different frequency bands (lines 497-500): “Considering the role of prestimulus oscillatory activity, future studies could further investigate the contribution of specific frequency bands to the unconscious and conscious stages involved in SQM processing, as the present analyses focused exclusively on ERP-based decoding.”
25. Line 639: The ability to decode V-AV versus NV does not imply that the integrated percept still has a slight offset. The two stimuli are completely different.

    As per our responses on several points above, we agree and have now removed this part from the Methods section, while revised the text in several other places to clarify this point (see our responses above to points 7, 15, 16, 19).
26. Line 641: There is no direct behavioral evidence here that participants consciously perceived an offset in the V-AV conditions.

    We removed this statement and improved the paragraph (lines 659-668). See also previous responses (e.g., points 7, 15 and 16).
27. Line 643: Conversely, these three conditions likely deliver slightly different consciously perceived offsets and the decoder could be sensitive to this.

    We agree (see point 26).

Supplementary Material

28. Line 1057: Cross-condition generalization from V vs NV to V-AV vs NV does not show that a single vernier is consciously perceived in V-AV. It only shows that offset(s) (as a general concept) are represented similarly in V and V-AV conditions.

    We clarified the sentence (lines 1171-1175): “The cross-condition results successfully decoded V-AV conditions from the NV condition in all cases (AUC above 0.5, one-tailed cluster-based permutation test, *p* < .05; Supplementary Figure S1C). These results confirm that the EEG patterns related to the V-AV and NV conditions differ (see also Figure 4A) and that the neural representation of a vernier is preserved even when two opposite verniers are presented (see also Figure 4F).”

## Reviewer #3

The authors use a clever paradigm and analysis approach to identify two stages of visual processing linked with prolonged unconscious integration and the establishment of a final conscious percept. The experimental design and EEG analyses appear to be carefully executed and the data meet high standards of quality. The paper is well written and has the potential to make a substantial contribution to the literature on this topic. This was truly an exciting study and a pleasure to read. I would recommend accepting this for publication after the authors consider making some minor revisions, including running a possible additional set of analysis as suggested below.

Respectfully signed,
Michael Pitts

We thank the Reviewer for the positive evaluation of our work and for providing important comments, which we are now addressing.

1. My primary concern is with how the authors describe the V-AV condition. It is not clear what subjects actually perceive in this condition. Do they perceive both vernier offsets, but confuse their order, or do they only perceive one of the offsets (sometimes the first and sometimes the second), or do they perceive no offset? All three of these possibilities would result in the same behavioral results (at-chance performance on discrimination of the first vernier in the stream). The authors should be more explicit about what subjects perceive in this condition and present evidence supporting one of these three possibilities.

   We thank the Reviewer for this point and apologize for the lack of clarity. In response to this and related comments raised by the other Reviewers, we have revised the manuscript throughout to clarify that, with the current forced-choice paradigm, we cannot directly infer the exact percept experienced in the V–AV condition. Specifically, participants may perceive either a fully integrated offset, resulting in the percept of a straight line, or a weaker residual offset arising from non-uniform integration, as already suggested in a previous study (Menétrey et al., 2023; see lines 194-198, 288-296) (see also our responses to points 7, and 16 from Reviewer #2).

We now explicitly acknowledge this limitation in the revised Discussion section (line 484-497). Importantly, however, this issue does not affect the main conclusions of the study, as our central findings concern the decodable neural representations of individual vernier events prior to their integration into the final percept.

2. For example, in the introduction, the authors write, "When the stream contains two vernier stimuli with opposite offsets, the offsets integrate mandatorily". This gives the impression that the anti-vernier offset cancels out the vernier offset leading subjects to integrate them and perceive no offset, thus leading to their at-chance performance in the V-AV conditions. However, the authors then write, "Instead, they perceive only a single offset", but they do not summarize the evidence for this.

   We removed the claim that "a single offset in perceived" both in the abstract (lines 30-32) and in the introduction (60-61).
3. In the results section, they write, "The key question that follows is whether the two EEG activation patterns observed reflect the conscious perception of a single vernier throughout the stream, or instead capture successive stages in which each individual vernier is first processed unconsciously and later integrated into a unified percept. To address this question, we tested whether the two V-AV conditions, where the second vernier occurred at different times (V0-AV2 vs. V0-AV4), could be decoded from V0 trials, where only a single vernier was shown and consciously perceived. Significant decoding in this comparison would indicate neural correlates of the second vernier offset that was processed unconsciously and integrated with the first vernier." I don't understand what they mean by the second vernier offset being "integrated" with the first. Again, is the second offset perceived? Integration implies that it's combined in some way, but if subjects only perceive the first one, isn't it more like the second one gets blocked or masked? And if they only perceive the first one, why are they at chance behaviorally?

   By “integration”, we mean that both vernier offsets are combined, resulting in a single integrated offset perceived throughout the entire stream, but this process does not necessarily result in perfect cancellation (i.e., a straight line). Instead, the two offsets contribute to a single, unified perceptual outcome. Within this framework, the second vernier is not consciously accessible as an independent element, nor is it simply masked; rather, it influences the final percept without being individually reportable. Chance-level performance can be explained by the fact that the perceived offset is equally likely to be congruent with either the first or the second vernier, as also suggested in previous work (Menétrey et al., 2023, see also response to point 1).

   Although we do not have direct behavioral evidence showing that a single vernier, potentially with a weaker offset, is perceived in the V–AV conditions, our decoding results provide support for this interpretation, as we found topographies associated with both unconscious and conscious processing in these analyses (see lines 285-294). However, to clarify the Results section, we have revised the relevant passage and now present the idea that a single offset may still be perceived in the V–AV conditions as a hypothesis (lines 194-198): “In the V-AV conditions, behavioral performance was around 50% (Figure 1D). As previously suggested, this may reflect either that both offsets are fully integrated, resulting in the perception of a straight line as in the NV condition (Drissi-Daoudi et al., 2019), or that there is an equal probability that one of the two vernier offsets ”wins” the integration process in a given trial (Menétrey et al., 2023).
4. The reason this is so important is that the authors potentially have 2 conditions which differ physically (1 vs. 2 vernier offsets presented on screen), but are equivalent perceptually (only 1 vernier offset is perceived). Thus, contrasting these two conditions can go a long way to distinguish unconscious from conscious processing. From the data presented, however, it's not clear whether subjects perceive 0, 1, or 2 vernier offsets in the V-AV conditions. I suggest clarifying the evidence for what subjects perceive in this condition, and adjusting some of the writing (e.g., "obligatorily integrated") to avoid confusing the reader on this important aspect of their study.

   We removed the terms “obligatorily” and "mandatorily" in all the manuscript and, as mentioned above, we added a section acknowledging that, due to the use of a forced-choice paradigm, the present study cannot determine from behavioral data whether participants perceived no offset or a single offset, or whether participants were indeed never able to segregate the two offsets in the V–AV condition (lines 484-497).
5. Related to this last point, the authors have other pairs of conditions that differ physically, but are the exact same perceptually: V0 vs. V2, V0 vs. V4, and V2 vs. V4. However, they do not report results from any analyses in which decoders were trained and tested on these distinctions. Wouldn't this be a cleaner set of contrasts to get at the same question they addressed with the V0 vs. V-AV analyses reported in Fig. 5? In other words, shouldn't we expect to see the first EEG effect but not the second, if the first is related to unconscious processing (based on physical stimulus differences) and the second is related to the final conscious percept (which does not differ across V0 vs. V2 vs. V4 conditions)? The lack of reporting on these three pairs of contrasts stands-out. I would strongly encourage the authors to run these analyses and add the results to the revised manuscript.

   We thank the Reviewer for this important suggestion, which was also in line with requests from the other Reviewers. We have now performed these additional analyses (V0 vs. V2, V0 vs. V4, and V2 vs. V4) and report them in the Supplementary Materials for the sake of space, while retaining the cross-condition decoding analyses in the main text.
   As expected, these additional analyses confirmed the dominant contribution of the occipital topography within the significant decoding windows between these comparisons, with little contribution from the later parietal topography (Supplementary Material: lines 1060-1091 and Supplementary Figure 1).
6. In Figure 5, panel C, the correlation results are only plotted out to 700ms, while in all other figures, data are shown going out to 1000ms. It is important to show this data out to 1000ms b/c the key finding here is the lack of the second effect. Perhaps the second effect was there but was delayed in these contrasts? (hints of it can be seen, starting around 650ms in the V0-AV2 vs. V0 and V0-AV4 vs. V0 plots).

   The time windows shown in Figure 5C, as well as in 6C, were restricted to the significant decoding windows identified in the main analyses (Figures 5A and 6B, respectively). We therefore felt it would not be appropriate to interpret patterns emerging outside these significant windows by performing additional significance tests beyond the time range defined by the original decoding results.
   Nevertheless, following the Reviewer’s suggestion, we now report the ERP plots for these comparisons across the full time window in the Supplementary Materials (Supplementary Material: lines 1212-1262, Supplementary Figure 4). These additional plots further show that the reported effects were confined to the specific temporal windows identified by the decoding analyses.
7. It would be helpful to add something about the task to the methods figures in Fig 1 A, B, or C. Visually depicting what the subjects were tasked with doing on each trial, and when they were supposed to do it, would help clarify the paradigm beyond the description in the methods section of the text.

   We have completed the illustration in Fig. 1A.
8. It was interesting to see that the first EEG effect was right lateralized (ipsilateral to the location of the attended part of the stimulus). Have the authors run any subjects on the same experiment but with the vernier offsets (and subjects' attention) being on the left? If not, this could make for a nice follow-up study to further investigate the meaning of the right-lateralized effect (this could be worth mentioning in the discussion when they note the unexpected laterality).

   We agree with Reviewer's comment and added it to the Discussion (lines 375-376): “...and future studies should assess the effect of attending to the left versus the right stream, as participants in the present study attended only to the right stream.”
9. The topography of the second EEG effect looks very similar to a typical P3b topography, and as the authors note in the discussion, because this was a trial-by-trial report paradigm, it's possible that this effect is related to performing the task rather than consciously perceiving the vernier offsets. Have the authors tried any no-report versions of this paradigm? It seems ideally suited for no-report, as perceiving vs. not-perceiving a vernier offset can be objectively manipulated, e.g., the same contrasts between V0 vs. NV; V2 vs. NV; V4 vs. NV, can all be done without having to ask subjects to report what they see on each trial (behavior can be validated separately in a report condition like they used here). I am not suggesting this is needed for the current paper, but I hope the authors will consider running such an experiment as this should eliminate the second EEG effect and potentially allow them to identify the genuine neural signals of the second stage that is associated with consciously perceiving the vernier offset.

   We never tried a no-report version of the SQM paradigm. However, this would be straightforward to implement and represents an interesting direction for future work. We now mention it in the Discussion (lines 400-402): “Nevertheless, a post-perceptual interpretation remains possible, and future studies incorporating no-report paradigms will be important to further clarify the role of this component.”
