# Supplementary material for: Sequential neural dynamics underlie unconscious integration and conscious perception of visual stimuli
Source: PLoS Biol. 2026 Jul 6;24(7):e3003894. doi: 10.1371/journal.pbio.3003894 (PMC13362400; doi:10.1371/journal.pbio.3003894)
Supplement: Peer review history

# Editor report: Decision Letter 0

**DOI:** 10.1371/journal.pbio.3003894.r001

**Authors:** Christian Schnell, PhD

4 Mar 2026

Dear Dr Menétrey,

Thank you for submitting your manuscript entitled "Neural evidence for a two-stage model of visual processing" for consideration as a Research Article by PLOS Biology.

Your manuscript has now been evaluated by the PLOS Biology editorial staff as well as by an academic editor with relevant expertise and I am writing to let you know that we would like to send your submission out for external peer review.

However, before we can send your manuscript to reviewers, we need you to complete your submission by providing the metadata that is required for full assessment. To this end, please login to Editorial Manager where you will find the paper in the 'Submissions Needing Revisions' folder on your homepage. Please click 'Revise Submission' from the Action Links and complete all additional questions in the submission questionnaire.

Once your full submission is complete, your paper will undergo a series of checks in preparation for peer review. After your manuscript has passed the checks it will be sent out for review. To provide the metadata for your submission, please Login to Editorial Manager (https://www.editorialmanager.com/pbiology) within two working days, i.e. by Mar 06 2026 11:59PM.

If your manuscript has been previously peer-reviewed at another journal, PLOS Biology is willing to work with those reviews in order to avoid re-starting the process. Submission of the previous reviews is entirely optional and our ability to use them effectively will depend on the willingness of the previous journal to confirm the content of the reports and share the reviewer identities. Please note that we reserve the right to invite additional reviewers if we consider that additional/independent reviewers are needed, although we aim to avoid this as far as possible. In our experience, working with previous reviews does save time.

If you would like us to consider previous reviewer reports, please edit your cover letter to let us know and include the name of the journal where the work was previously considered and the manuscript ID it was given. In addition, please upload a response to the reviews as a 'Prior Peer Review' file type, which should include the reports in full and a point-by-point reply detailing how you have or plan to address the reviewers' concerns.

During the process of completing your manuscript submission, you will be invited to opt-in to posting your pre-review manuscript as a bioRxiv preprint. Visit http://journals.plos.org/plosbiology/s/preprints for full details. If you consent to posting your current manuscript as a preprint, please upload a single Preprint PDF.

Feel free to email us at plosbiology@plos.org if you have any queries relating to your submission.

Kind regards,

Christian

Christian Schnell, PhD

Senior Editor

PLOS Biology

cschnell@plos.org

# Peer review: Decision Letter 1

**DOI:** 10.1371/journal.pbio.3003894.r002

**Authors:** Christian Schnell, PhD, Christian Schnell, PhD

10 Apr 2026

Dear Dr Menétrey,

Thank you for your patience while your manuscript "Neural evidence for a two-stage model of visual processing" went through peer-review at PLOS Biology. Your manuscript has now been evaluated by the PLOS Biology editors, an Academic Editor with relevant expertise, and by several independent reviewers.

In light of the reviews, which you will find at the end of this email, we are pleased to offer you the opportunity to address the comments from the reviewers in a revision that we anticipate should not take you very long. We will then assess your revised manuscript and your response to the reviewers' comments with our Academic Editor aiming to avoid further rounds of peer-review, although we might need to consult with the reviewers, depending on the nature of the revisions.

In addition to these revisions, you may need to complete some formatting changes, which you will receive in a follow up email. A member of our team will be in touch with a set of requests shortly. If you do not receive a separate email within a few days, please assume that checks have been completed, and no additional changes are required.

We expect to receive your revised manuscript at the end of next month. Please email us (plosbiology@plos.org) if you have any questions or concerns, or would like to request an extension.

At this stage, your manuscript remains formally under active consideration at our journal; please notify us by email if you do not intend to submit a revision so that we withdraw the manuscript.

**IMPORTANT - SUBMITTING YOUR REVISION**

Your revisions should address the specific points made by each reviewer. Please submit the following files along with your revised manuscript:

1. A 'Response to Reviewers' file - this should detail your responses to the editorial requests, present a point-by-point response to all of the reviewers' comments, and indicate the changes made to the manuscript.

*NOTE: In your point-by-point response to the reviewers, please provide the full context of each review. Do not selectively quote paragraphs or sentences to reply to. The entire set of reviewer comments should be present in full and each specific point should be responded to individually.

You should also cite any additional relevant literature that has been published since the original submission and mention any additional citations in your response.

2. In addition to a clean copy of the manuscript, please also upload a 'track-changes' version of your manuscript that specifies the edits made. This should be uploaded as a "Revised Article with Changes Highlighted " file type.

*Resubmission Checklist*

When you are ready to resubmit your revised manuscript, please refer to this resubmission checklist: https://plos.io/Biology_Checklist

To submit a revised version of your manuscript, please go to https://www.editorialmanager.com/pbiology/ and log in as an Author. Click the link labelled 'Submissions Needing Revision' where you will find your submission record.

Please make sure to read the following important policies and guidelines while preparing your revision:

*Published Peer Review*

Please note while forming your response, if your article is accepted, you may have the opportunity to make the peer review history publicly available. The record will include editor decision letters (with reviews) and your responses to reviewer comments. If eligible, we will contact you to opt in or out. Please see here for more details:

https://blogs.plos.org/plos/2019/05/plos-journals-now-open-for-published-peer-review/

*PLOS Data Policy*

Please note that as a condition of publication PLOS' data policy (http://journals.plos.org/plosbiology/s/data-availability) requires that you make available all data used to draw the conclusions arrived at in your manuscript. If you have not already done so, you must include any data used in your manuscript either in appropriate repositories, within the body of the manuscript, or as supporting information (N.B. this includes any numerical values that were used to generate graphs, histograms etc.). For an example see here: http://www.plosbiology.org/article/info%3Adoi%2F10.1371%2Fjournal.pbio.1001908#s5

*Blot and Gel Data Policy*

We require the original, uncropped and minimally adjusted images supporting all blot and gel results reported in an article's figures or Supporting Information files. We will require these files before a manuscript can be accepted so please prepare them now, if you have not already uploaded them. Please carefully read our guidelines for how to prepare and upload this data: https://journals.plos.org/plosbiology/s/figures#loc-blot-and-gel-reporting-requirements

*Protocols deposition*

To enhance the reproducibility of your results, we recommend that if applicable you deposit your laboratory protocols in protocols.io, where a protocol can be assigned its own identifier (DOI) such that it can be cited independently in the future. Additionally, PLOS ONE offers an option for publishing peer-reviewed Lab Protocol articles, which describe protocols hosted on protocols.io. Read more information on sharing protocols at https://plos.org/protocols?utm_medium=editorial-email&utm_source=authorletters&utm_campaign=protocols

Thank you again for your submission to our journal. We hope that our editorial process has been constructive thus far, and we welcome your feedback at any time. Please don't hesitate to contact us if you have any questions or comments.

Sincerely,

Christian

Christian Schnell, PhD

Senior Editor

PLOS Biology

cschnell@plos.org

----------------------------------------------------------------

REVIEWS:

Reviewer #1: Review

Menétrey et al. "Neural evidence for a two-stage model of visual processing"

Comments

The paper reports the results of an EEG decoding analysis of the interesting, as well as slightly vexing, SQM paradigm. The study has been expertly conducted, and the results are rather beautiful. The conclusions drawn also seem clear and appropriate, and as such I have little to complain overall. Nevertheless, I still have a few specific points/queries:

Decoding in both the V (0, 2, and 4) the V-AV (2 and 4) conditions showed that the presence of the (last) Vernier element was delayed as its onset occurred later in the stream. This is a nice result, but I noticed the delay is not constant. Between V0 and V2, there is about 90 ms, close to the actual 100 ms SOA difference, but between 2 and 4, the delay is only 40 ms. What could be the reason for this? Might it be some kind of perceptual acceleration, caused by the succession of stimulus displays, like we found in the missing element task (Wolff & Akyurek, in press)? I'd be interested in the authors' take on this.

Scholte et al. have recently claimed that binding (=integration?) does not exist in the brain, or is at least not needed conceptually. I thought it may be interesting to consider this idea in the light of the present results, which to me at least seem to suggest the opposite. I do apologize in advance if this only leads to a garden path. https://doi.org/10.1016/j.tics.2025.03.002

In the discussion, reference is made to the N170/VAN component of the ERP. It might be worthwhile to compute and actually show this ERP from the present data (time-locked to the Vernier), rather than only the decoding scores and their topography, to further substantiate this comparison.

Signed,

Elkan Akyürek

Reviewer #2 (Max Levinson also signed his report): The manuscript "Neural evidence for a two-stage model of visual processing" by Menétrey et al. is an elegant demonstration of the two-stage model for unconscious/conscious processing espoused by Herzog and colleagues over the past decade. The results are striking and nicely contextualized within this framework. They find that neural activity during the sequential metacontrast task (SQM) consists of two temporally-distinct stages: one dominated by occipital activity corresponding to unconscious processing of vernier offsets, followed by a parietal topography corresponding to conscious perception. The paper has clear implications for the broader fields of perception and consciousness.

Although the findings largely support the two-stage model, the manuscript is written as if this model was already established fact. The authors should soften these claims, especially as the purpose of this study is to discover two neural stages that would support the theoretical model. Similarly, claims about the phenomenology of SQM are made without behavioral evidence, such as that participants never perceive two offsets in V-AV conditions. Some more caution throughout the manuscript would improve its readability and, I think, better demonstrate the novelty of these interesting and likely very impactful findings.

Specific comments follow below.

Abstract

1.The first sentence already begs the question of the two-stage model hypothesis. Postdictive effects do not necessarily require periods of unconscious processing if, for example, consciousness is continuously updated and we simply forget previous experiences.

2.Line 33: Similarly, my understanding is that the purpose of this experiment is to use EEG to discover whether there are two subsequent stages that correspond to unconscious and conscious processing. But here it is already claimed that the stages are "well separated in time" in the SQM, presumably concluded from prior behavioral data. However, if our conscious experience is of an integrated offset from the beginning until the end of the trial, then conscious and unconscious processing appear to fully overlap in time.

Introduction

3.Lines 54-55: This sentence was a bit confusing because "lines" is supposed to refer to the vertical pairs of lines at each stimulus increment, but I initially read it as referring to each individual line segment. (Each individual line is always vertically straight). Maybe using a term like "line pair" would be helpful.

4.Line 65: I suggest explicitly addressing the potential role of motion for producing such long windows in the SQM. Non-moving visual stimuli might be integrated over shorter windows.

5.Lines 87-90: unclear if this "rationale" is a hypothesis about how the SQM works, or findings from previous behavioral studies (if so, they should be cited).

6.Fig. 1D: I recommend removing the colored backgrounds in the bar plots - they are unnecessary and make the individual dots hard to see.

7.Fig. 1D legend: here it is claimed that V-AV performance is at 50% because participants could not perceive the individual offsets, but later in the paper it is claimed that perception is biased towards one or the other offset in each individual trial.

Results

8.Line 160: A large mean effect size does not necessarily imply consistency across participants. Better would be to report the prevalence of the effect in the sample, such as the percentage of participants showing the effect in the same direction.

9.Lines 161-163: Negative values in the first topography and positive values in the second topography are both labeled as "stronger activation" or "increased activation."

10.Fig. 3A, bottom: Cohen's d is labeled as d' in many figures.

11.Fig. 3C: is it relevant that the two topographies are anti-correlated? (as opposed to one map's r staying at 0 while the other significantly increases)

12.Line 175: I am not sure what the "difference between each activation pattern and zero" means. Is it the average activation in each electrode, or some measure of the topographical pattern?

13.Line 186: "Location" is unclear, as it could refer to the increment in the SQM sequence (temporal location), the horizontal position of the offset, or the left versus right streams. From the context of the sentence I believe it is referring to the temporal location. If so, what is the evidence that the temporal location was not consciously perceived?

14.Furthermore, none of the results so far show decoding of the temporal location of the vernier (e.g., V2 vs V4). There is only decoding of one temporal location versus no vernier (e.g. V2 vs NV).

15.Line 199: This result does not appear related to the weak integration hypothesis from the previous sentence. One would expect to decode V-AV from NV trials even if the two offsets are perfectly integrated unconsciously (so no offset is perceived).

16.Line 207: In general, the article is missing more a convincing argument that the two offsets are always mandatorily integrated in conscious perception. This claim is made several times, sometimes with citations to previous work, but it is not tested in the present study (the participants are not asked whether they perceive one or two offsets). It would be helpful to add some more explanation for how the cited studies show that the two offsets are always integrated into one.

17.In addition, if the two offsets are not perfectly integrated and subjects still perceive a slight offset, V-AV versus NV decoding could pick up differences in conscious processing but not unconscious processing.

18.Lines 248 and 253: same concern that subjects were not asked if they perceived a second offset or not.

19.Line 251: V0-AV2 and V0-AV4 may weigh the two offsets differently and produce different conscious experiences of the integrated offset. Fig. 1D suggests this may be the case. The decoder could be picking up the difference in conscious perception, not the temporal position of the second vernier.

20.Line 256: I suggest labeling the first topography as "occipital" and the second as "parietal", or something like that, to help orient the reader when discussing the two topographies.

21.Lines 267 and 321: There is no temporal generalization in figures 5 and 6.

22.Line 297: How were the offset directions and button mappings randomized / counterbalanced? Is there any risk that the correct v. incorrect decoder is picking up either unconscious offset direction or motor signals?

23.I imagine an offset might be missed if the eyes are moving during its brief presentation (27.8 ms), and eye movement could therefore account for correct vs. incorrect decoding. Participants were instructed to covertly attend but, to my understanding, eye position was not monitored. It would be informative to check if the EOG data differentiate correct from incorrect trials, particularly during 1) the offset presentation, 2) the periods of significant correct vs. incorrect decoding.

Materials and Methods

24.Why was 40 Hz chosen for the lowpass? This precludes detecting gamma-range activity that is often considered a medium for unconscious visual processing.

25.Line 639: The ability to decode V-AV versus NV does not imply that the integrated percept still has a slight offset. The two stimuli are completely different.

26.Line 641: There is no direct behavioral evidence here that participants consciously perceived an offset in the V-AV conditions.

27.Line 643: Conversely, these three conditions likely deliver slightly different consciously perceived offsets and the decoder could be sensitive to this.

Supplementary Material

28.Line 1057: Cross-condition generalization from V vs NV to V-AV vs NV does not show that a single vernier is consciously perceived in V-AV. It only shows that offset(s) (as a general concept) are represented similarly in V and V-AV conditions.

Reviewer #3: The authors use a clever paradigm and analysis approach to identify two stages of visual processing linked with prolonged unconscious integration and the establishment of a final conscious percept. The experimental design and EEG analyses appear to be carefully executed and the data meet high standards of quality. The paper is well written and has the potential to make a substantial contribution to the literature on this topic. This was truly an exciting study and a pleasure to read. I would recommend accepting this for publication after the authors consider making some minor revisions, including running a possible additional set of analysis as suggested below.

Respectfully signed,

Michael Pitts

1. My primary concern is with how the authors describe the V-AV condition. It is not clear what subjects actually perceive in this condition. Do they perceive both vernier offsets, but confuse their order, or do they only perceive one of the offsets (sometimes the first and sometimes the second), or do they perceive no offset? All three of these possibilities would result in the same behavioral results (at-chance performance on discrimination of the first vernier in the stream). The authors should be more explicit about what subjects perceive in this condition and present evidence supporting one of these three possibilities.

For example, in the introduction, the authors write, "When the stream contains two vernier stimuli with opposite offsets, the offsets integrate mandatorily". This gives the impression that the anti-vernier offset cancels out the vernier offset leading subjects to integrate them and perceive no offset, thus leading to their at-chance performance in the V-AV conditions. However, the authors then write, "Instead, they perceive only a single offset", but they do not summarize the evidence for this.

In the results section, they write, "The key question that follows is whether the two EEG activation patterns observed reflect the conscious perception of a single vernier throughout the stream, or instead capture successive stages in which each individual vernier is first processed unconsciously and later integrated into a unified percept. To address this question, we tested whether the two V-AV conditions, where the second vernier occurred at different times (V0-AV2 vs. V0-AV4), could be decoded from V0 trials, where only a single vernier was shown and consciously perceived. Significant decoding in this comparison would indicate neural correlates of the second vernier offset that was processed unconsciously and integrated with the first vernier." I don't understand what they mean by the second vernier offset being "integrated" with the first. Again, is the second offset perceived? Integration implies that it's combined in some way, but if subjects only perceive the first one, isn't it more like the second one gets blocked or masked? And if they only perceive the first one, why are they at chance behaviorally?

The reason this is so important is that the authors potentially have 2 conditions which differ physically (1 vs. 2 vernier offsets presented on screen), but are equivalent perceptually (only 1 vernier offset is perceived). Thus, contrasting these two conditions can go a long way to distinguish unconscious from conscious processing. From the data presented, however, it's not clear whether subjects perceive 0, 1, or 2 vernier offsets in the V-AV conditions. I suggest clarifying the evidence for what subjects perceive in this condition, and adjusting some of the writing (e.g., "obligatorily integrated") to avoid confusing the reader on this important aspect of their study.

2. Related to this last point, the authors have other pairs of conditions that differ physically, but are the exact same perceptually: V0 vs. V2, V0 vs. V4, and V2 vs. V4. However, they do not report results from any analyses in which decoders were trained and tested on these distinctions. Wouldn't this be a cleaner set of contrasts to get at the same question they addressed with the V0 vs. V-AV analyses reported in Fig. 5? In other words, shouldn't we expect to see the first EEG effect but not the second, if the first is related to unconscious processing (based on physical stimulus differences) and the second is related to the final conscious percept (which does not differ across V0 vs. V2 vs. V4 conditions)? The lack of reporting on these three pairs of contrasts stands-out. I would strongly encourage the authors to run these analyses and add the results to the revised manuscript.

3. In Figure 5, panel C, the correlation results are only plotted out to 700ms, while in all other figures, data are shown going out to 1000ms. It is important to show this data out to 1000ms b/c the key finding here is the lack of the second effect. Perhaps the second effect was there but was delayed in these contrasts? (hints of it can be seen, starting around 650ms in the V0-AV2 vs. V0 and V0-AV4 vs. V0 plots).

4. It would be helpful to add something about the task to the methods figures in Fig 1 A, B, or C. Visually depicting what the subjects were tasked with doing on each trial, and when they were supposed to do it, would help clarify the paradigm beyond the description in the methods section of the text.

5. It was interesting to see that the first EEG effect was right lateralized (ipsilateral to the location of the attended part of the stimulus). Have the authors run any subjects on the same experiment but with the vernier offsets (and subjects' attention) being on the left? If not, this could make for a nice follow-up study to further investigate the meaning of the right-lateralized effect (this could be worth mentioning in the discussion when they note the unexpected laterality).

6. The topography of the second EEG effect looks very similar to a typical P3b topography, and as the authors note in the discussion, because this was a trial-by-trial report paradigm, it's possible that this effect is related to performing the task rather than consciously perceiving the vernier offsets. Have the authors tried any no-report versions of this paradigm? It seems ideally suited for no-report, as perceiving vs. not-perceiving a vernier offset can be objectively manipulated, e.g., the same contrasts between V0 vs. NV; V2 vs. NV; V4 vs. NV, can all be done without having to ask subjects to report what they see on each trial (behavior can be validated separately in a report condition like they used here). I am not suggesting this is needed for the current paper, but I hope the authors will consider running such an experiment as this should eliminate the second EEG effect and potentially allow them to identify the genuine neural signals of the second stage that is associated with consciously perceiving the vernier offset.

# Author comment: Author response to Decision Letter 2

**DOI:** 10.1371/journal.pbio.3003894.r003

28 May 2026

Attachment Submitted filename: PlosBiol_Reply_R1.docx

# Peer review: Decision Letter 2

**DOI:** 10.1371/journal.pbio.3003894.r004

**Authors:** Christian Schnell, PhD, Christian Schnell, PhD, Christian Schnell, PhD

16 Jun 2026

Dear Dr Menétrey,

Thank you for your patience while we considered your revised manuscript "Neural evidence for a two-stage model of visual processing" for publication as a Research Article at PLOS Biology. This revised version of your manuscript has been evaluated by the PLOS Biology editors, the Academic Editor and two the original reviewers.

Based on the reviews and on our Academic Editor's assessment of your revision, we are likely to accept this manuscript for publication, provided you satisfactorily address the following data and other policy-related requests:

* We would like to suggest a different title to improve its accessibility for our broad audience:

Sequential neural dynamics underlie unconscious integration and conscious perception of visual stimuli

* Please note that per journal policy, the model system/species studied should be clearly stated in the abstract of your manuscript.

* Please move the references from the supplementary information file to the main reference list, or at least make sure that those references are listed there as well.

* Please move methodological details or supplementary results from the supplementary information to the main manuscript file. We do not have a word count limit and want to make it as straightforward as possible for readers to find the information they need.

* DATA POLICY:

You may be aware of the PLOS Data Policy, which requires that all data be made available without restriction: http://journals.plos.org/plosbiology/s/data-availability. For more information, please also see this editorial: http://dx.doi.org/10.1371/journal.pbio.1001797

Note that we do not require all raw data. Rather, we ask that all individual quantitative observations that underlie the data summarized in the figures and results of your paper be made available in one of the following forms:

1) Supplementary files (e.g., excel). Please ensure that all data files are uploaded as 'Supporting Information' and are invariably referred to (in the manuscript, figure legends, and the Description field when uploading your files) using the following format verbatim: S1 Data, S2 Data, etc. Multiple panels of a single or even several figures can be included as multiple sheets in one excel file that is saved using exactly the following convention: S1_Data.xlsx (using an underscore).

2) Deposition in a publicly available repository. Please also provide the accession code or a reviewer link so that we may view your data before publication.

Regardless of the method selected, please ensure that you provide the individual numerical values that underlie the summary data displayed in the following figure panels as they are essential for readers to assess your analysis and to reproduce it: 1D

NOTE: the numerical data provided should include all replicates AND the way in which the plotted mean and errors were derived (it should not present only the mean/average values).

Please also ensure that figure legends in your manuscript include information on where the underlying data can be found, and ensure your supplemental data file/s has a legend.

Please ensure that your Data Statement in the submission system accurately describes where your data can be found.

* CODE POLICY

Per journal policy, if you have generated any custom code during the course of this investigation, please make it available without restrictions. Please ensure that the code is sufficiently well documented and reusable, and that your Data Statement in the Editorial Manager submission system accurately describes where your code can be found. More information on our Code Policy, what and how to share can be found here: https://journals.plos.org/plosbiology/s/code-availability

Please note that we cannot accept sole deposition of code in GitHub, as this could be changed after publication. However, you can archive this version of your publicly available GitHub code to Zenodo. Once you do this, it will generate a DOI number, which you will need to provide in the Data Accessibility Statement (you are welcome to also provide the GitHub access information). See the process for doing this here: https://docs.github.com/en/repositories/archiving-a-github-repository/referencing-and-citing-content

As you address these items, please take this last chance to review your reference list to ensure that it is complete and correct. If you have cited papers that have been retracted, please include the rationale for doing so in the manuscript text, or remove these references and replace them with relevant current references. Any changes to the reference list should be mentioned in the cover letter that accompanies your revised manuscript.

In addition to these revisions, you may need to complete some formatting changes, which you will receive in a follow up email. A member of our team will be in touch with a set of requests shortly. If you do not receive a separate email within a few days, please assume that checks have been completed, and no additional changes are required.

We expect to receive your revised manuscript within two weeks.

To submit your revision, please go to https://www.editorialmanager.com/pbiology/ and log in as an Author. Click the link labelled 'Submissions Needing Revision' to find your submission record. Your revised submission must include the following:

- a cover letter that should detail your responses to any editorial requests, if applicable, and whether changes have been made to the reference list

- a Response to Reviewers file that provides a detailed response to the reviewers' comments (if applicable, if not applicable please do not delete your existing 'Response to Reviewers' file.)

- a track-changes file indicating any changes that you have made to the manuscript.

NOTE: If Supporting Information files are included with your article, note that these are not copyedited and will be published as they are submitted. Please ensure that these files are legible and of high quality (at least 300 dpi) in an easily accessible file format. For this reason, please be aware that any references listed in an SI file will not be indexed. For more information, see our Supporting Information guidelines:

https://journals.plos.org/plosbiology/s/supporting-information

*Published Peer Review History*

Please note that you may have the opportunity to make the peer review history publicly available. The record will include editor decision letters (with reviews) and your responses to reviewer comments. If eligible, we will contact you to opt in or out. Please see here for more details:

https://plos.org/published-peer-review-history/

*Press*

Should you, your institution's press office or the journal office choose to press release your paper, please ensure you have opted out of Early Article Posting on the submission form. We ask that you notify us as soon as possible if you or your institution is planning to press release the article.

*Protocols deposition*

To enhance the reproducibility of your results, we recommend that if applicable you deposit your laboratory protocols in protocols.io, where a protocol can be assigned its own identifier (DOI) such that it can be cited independently in the future. Additionally, PLOS ONE offers an option for publishing peer-reviewed Lab Protocol articles, which describe protocols hosted on protocols.io. Read more information on sharing protocols at https://plos.org/protocols?utm_medium=editorial-email&utm_source=authorletters&utm_campaign=protocols

Please do not hesitate to contact me should you have any questions.

Sincerely,

Christian

Christian Schnell, PhD

Senior Editor

cschnell@plos.org

PLOS Biology

------------------------------------------------------------------------

Reviewer remarks:

Reviewer #2 (Max Levinson signed his report): The authors have satisfactorily addressed all of my concerns. I look forward to seeing this important manuscript published!

Reviewer #3: The reviewers have addressed all of the comments from the first round of review. While this was already a great paper, the revised version is excellent.

# Editor report: Decision Letter 3

**DOI:** 10.1371/journal.pbio.3003894.r005

**Authors:** Christian Schnell, PhD, Christian Schnell, PhD, Christian Schnell, PhD, Christian Schnell, PhD

24 Jun 2026

Dear Dr Menétrey,

Thank you for the submission of your revised Research Article "Sequential neural dynamics underlie unconscious integration and conscious perception of visual stimuli" for publication in PLOS Biology. On behalf of my colleagues and the Academic Editor, Simon van Gaal, I am pleased to say that we can in principle accept your manuscript for publication, provided you address any remaining formatting and reporting issues. These will be detailed in an email you should receive within 2-3 business days from our colleagues in the journal operations team; no action is required from you until then. Please note that we will not be able to formally accept your manuscript and schedule it for publication until you have completed any requested changes.

Please take a minute to log into Editorial Manager at http://www.editorialmanager.com/pbiology/, click the "Update My Information" link at the top of the page, and update your user information to ensure an efficient production process.

PRESS

We frequently collaborate with press offices. If your institution or institutions have a press office, please notify them about your upcoming paper at this point, to enable them to help maximize its impact. If the press office is planning to promote your findings, we would be grateful if they could coordinate with biologypress@plos.org. If you have previously opted in to the early version process, we ask that you notify us immediately of any press plans so that we may opt out on your behalf.

We also ask that you take this opportunity to read our Embargo Policy regarding the discussion, promotion and media coverage of work that is yet to be published by PLOS. As your manuscript is not yet published, it is bound by the conditions of our Embargo Policy. Please be aware that this policy is in place both to ensure that any press coverage of your article is fully substantiated and to provide a direct link between such coverage and the published work. For full details of our Embargo Policy, please visit http://www.plos.org/about/media-inquiries/embargo-policy/.

Thank you again for choosing PLOS Biology for publication and supporting Open Access publishing. We look forward to publishing your study.

Sincerely,

Christian

Christian Schnell, PhD

Senior Editor

PLOS Biology

cschnell@plos.org
